# Patterns of restricted TCR usage following SARS-CoV-2 vaccination and severe disease

**DOI:** 10.3389/fimmu.2025.1576903

**Published:** 2025-10-02

**Authors:** Emily Parsons, Zhongyan Lu, Stephanie A. Richard, Amanda Zelkoski, Janifer Le, Naraen Palanikumar, Phuong Nguyen, Camille Alba, Gauthaman Sukumar, John Rosenberger, Xijun Zhang, Timothy H. Burgess, Rhonda Colombo, Katrin Mende, Catherine Berjohn, Nursat Epsi, Brian K. Agan, David Tribble, David A. Lindholm, Clifton L. Dalgard, Simon D. Pollett, Allison M. W. Malloy, EPICC COVID-19 Cohort Study Group

**Affiliations:** ^1^ Department of Pediatrics, Uniformed Services University of the Health Sciences, Bethesda, MD, United States; ^2^ Henry M. Jackson Foundation for the Advancement of Military Medicine, Inc., Bethesda, MD, United States; ^3^ Infectious Disease Clinical Research Program, Department of Preventive Medicine and Biostatistics, Uniformed Services University of the Health Sciences, Bethesda, MD, United States; ^4^ Department of Anatomy, Physiology, and Genetics, Uniformed Services University of the Health Sciences, Bethesda, MD, United States; ^5^ American Genome Center, Uniformed Services University of the Health Sciences, Bethesda, MD, United States; ^6^ Department of Medicine, Uniformed Services University of the Health Sciences, Bethesda, MD, United States; ^7^ Division of Infectious Diseases, Madigan Army Medical Center, Tacoma, WA, United States; ^8^ Brooke Army Medical Center, Joint Base San Antonio-Ft Sam Houston, TX, United States; ^9^ Division of Infectious Diseases, Naval Medical Center San Diego, San Diego, CA, United States

**Keywords:** SARS-CoV-2, CD8 T cells, T cell receptor sequencing, vaccination, T cell receptor (TCR)

## Abstract

**Introduction:**

T cells influence COVID-19 severity and establish long-lasting immune memory in response to vaccination and infection. The diversity of the T cell repertoire, and complexity of T cell epitope recognition, make it challenging to define protective epitope-specific T cells. In this study, we created a highly specific TCR meta-database to identify T cell epitopes from the nearly complete SARS-CoV-2 proteome and determine whether vaccination with mRNA vaccines influenced the TCR repertoire.

**Methods:**

Using this meta-database, we analyzed immunosequencing data of genomic DNA to define the variable region of T cell receptor (TCR) b chain (TCRB) sequences among participants in a longitudinal COVID-19 cohort study. The TCR repertoire was compared between participants who were vaccinated or unvaccinated against SARS-CoV-2 and stratified by disease severity. TCR diversity was measured using clonality, an index defined as the inverted normalized Shannon entropy.

**Results:**

Highly clonal TCR repertoires correlated with age and comorbidities. Using our meta-database approach, we found that vaccinated participants hospitalized with infection had the most restricted SARS-CoV-2-specific CD8 TCR repertoire. However, TCRB with predicted specificity to non-spike SARS-CoV-2 proteins dominated the response, even in vaccinated participants. We identified a peptide sequence in the ORF10 accessory protein that was more frequently recognized in study participants with mild disease. Conversely, CD8 T cell recognition of a peptide sequence in ORF1ab more closely correlated with severe disease.

**Discussion:**

Overarchingly, TCR repertoire analysis revealed that CD8 T cells responding to SARS-CoV-2 broadly recognize epitopes across the SARS-CoV-2 proteome, and provided opportunities to identify epitopes associated with disease.

## 1 Introduction

Early T cell responses have been associated with milder disease upon infection with SARS-CoV-2 (1–3), and robust convalescent T cell responses also correlated with milder symptoms (4–6). In murine models, lung-resident SARS-CoV-2-specific CD4 and CD8 T cells can provide effective protection, even in the absence of neutralizing antibodies (7). In addition, circulating SARS-CoV-2-specific CD4 and CD8 T cells are measurable for over a year after primary infection (8–10), and contribute to viral control in breakthrough infections (11, 12).

SARS-CoV-2 vaccines have been instrumental in decreasing the risk of severe disease (13–15). Messenger RNA (mRNA) vaccines, like the mRNA-1273 and BNT162b2 vaccines, encode for the SARS-CoV-2 spike (S) protein and induce antibodies and T cell responses against this viral protein (16–19). Antibody magnitude and neutralization are most frequently used as correlates of protection against severe COVID - 19, however titers decline 6 months post vaccination (20) and may not adequately control the development of variants of concern (VOC) (21, 22). T cell epitopes have been shown to be more conserved across VOC and T cells have been shown to provide cross-protection (23–25).

The T cell receptor (TCR) repertoire is tremendously diverse within and between individuals. T cells recognize pathogens through presentation of linear epitopes by major histocompatibility complexes (MHCs) that are polymorphic and result in broad peptide presentation that differs between individuals. Upon recognition of a peptide-MHC complex (pMHC) by a specific TCRs, naïve T cells begin differentiation and clonal expansion, resulting in detectable changes of the TCR repertoire specific to the antigen (26, 27). Therefore, TCR repertoires represent a functional signature of the T cell responses. Characterization of total, as well as SARS-CoV-2-specific, TCR repertoires can reveal associations with disease that may identify opportunities for vaccine design and therapeutic intervention.

The majority of human TCRs are composed of disulfide-linked α and β chains, each containing a variable and a constant domain (28). Somatic rearrangement of the variable (V), diversity (D) and joining (J) gene segments, together with random additions or deletions of nucleotides form the β chain (29). TCR diversity results from six variable hairpin loops located in the α/β variable domains, named complementarity-determining regions (CDRs). There are three α CDRs and three β CDRs (CDR1β, CDR2β, CDR3β). Recombination of the V, D, and J genes in the CDR3β and V and J genes in the CDR3α results in a high degree of diversity primarily driven by the CDR3β region. The diversity of the TCR repertoire, as well as the presence of TCRs cross-reactive to multiple epitopes, creates a significant challenge to defining pathogen-specific TCRs. SARS-CoV-2-specific TCRs have been identified based on a variety of techniques including *ex vivo* binding and *in vitro* responses to synthesized peptides, machine learning prediction methods, and approaches comparing healthy controls (30–35). Using these techniques, prior studies have shown that TCR repertoire diversity decreases with SARS-CoV-2 infection and disease severity (35–37). Conversely, SARS-CoV-2 infection in previously vaccinated individuals can broaden the SARS-CoV-2-specific TCR repertoire (12). Vaccine-induced expansion of epitope-specific T cells and their association with COVID - 19 disease severity is incompletely understood.

In this study, we investigated TCR repertoires upon SARS-CoV-2 infection and characterized differences in TCR recognition by disease severity and vaccination status. We focused our sequencing analysis on the CDR3β (TCRB), which is the most variable region, containing the diversity (D) gene segment, along with the V and J genes, using the ImmunoSEQ assay (Adaptive Biotechnologies) (38, 39). TCR epitope recognition was evaluated using over twenty-five published TCRB databases; including the ImmuneCODE database (Adaptive Biotechnologies) (40) and the VDJ database (41) in a novel approach to identify SARS-CoV-2-specific TCR sequences. By combining wet and dry lab analysis of the TCR response, we define patterns of TCR usage and specific epitope recognition after COVID - 19 mRNA vaccination and severe disease to provide insight for vaccines and therapeutics targeting T cells in those at highest risk.

## 2 Results

### 2.1 The clonality score of the total TCR repertoire correlated with age and comorbidity

**Figure 1:**
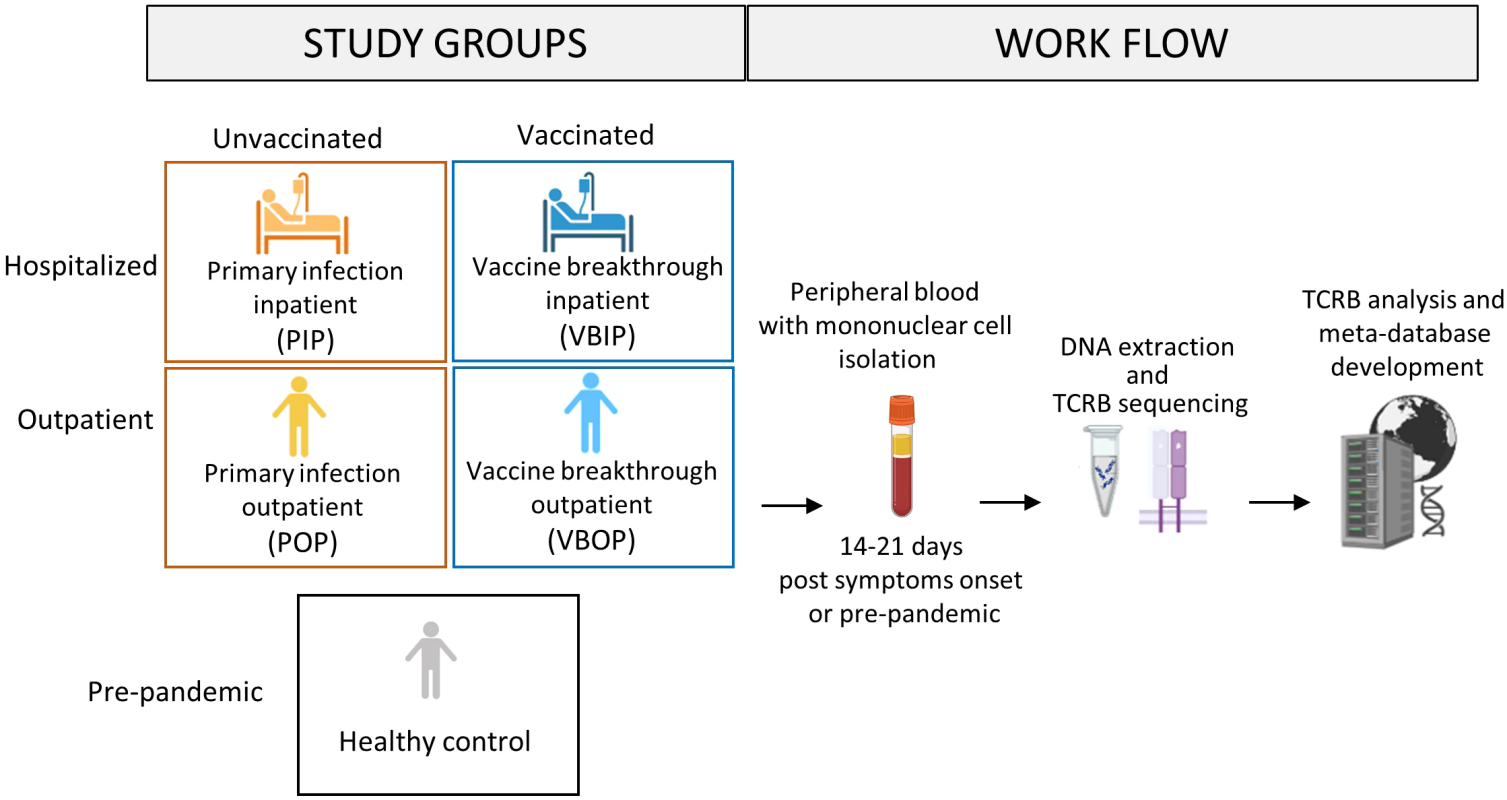
Schematic of study design. Peripheral blood was obtained from study participants with primary SARS-CoV-2 infection who had been either vaccinated or unvaccinated in the EPICC cohort. The TCRB chain was sequenced from extracted DNA from EPICC participants, as well as from cryopreserved PBMCs from pre-pandemic healthy donors. Multiple TCR databases from both pre-pandemic and SARS-CoV-2-specific studies were incorporated into the meta-database. Created in BioRender. Parsons, E. (2025) https://BioRender.com/3dibsyi.

TCR repertoire analysis was performed for a subset of participants enrolled in the Epidemiology, Immunology, and Clinical Characteristics of Emerging Infectious Diseases with Pandemic Potential (EPICC) study which enrolled participants tested for or who had a high-risk exposure to COVID - 19 from March 2020 to May 2022. The focused sub-analysis included herein analyzed a convenience sample of peripheral blood collected 14 – 21 days post symptom onset (DPSO) from participants who had a positive SARS-CoV-2 PCR. Disease was categorized as clinically mild (outpatient) or severe resulting in hospitalization (inpatient) (**Table 1**). Based on their vaccination record, the participants were also sub-divided into those with SARS-CoV-2 infection who had not received a COVID - 19 vaccine (primary infection group), and those who had been fully vaccinated (defined as having received two or more doses of a COVID - 19 S-based mRNA vaccine administered two or more weeks prior to infection) resulting in four study groups; primary infection inpatients (PIP), primary infection outpatients (POP), vaccine breakthrough infection inpatients (VBIP), and vaccine breakthrough infection outpatients (VBOP) (**Figure 1**). Sex was not significantly different among groups. The age range was 19 – 87 years. Outpatients were younger than inpatients (**Table 1**). Inpatients also had higher Charlson Comorbidity Index (CCI) scores (**Table 1**). Importantly, there were no significant differences in the DPSO peripheral blood collection between clinical study groups. TCRs were sequenced from peripheral blood mononuclear cells (PBMCs) of EPICC participants and 5 healthy adults whose peripheral blood was collected between August and September of 2019.

**Table 1:** Characteristics of participants included for TCR analysis.

Characteristic	Inpatient, unvaccinated (N = 11)	Outpatient, unvaccinated (N = 4)	Inpatient, vaccinated (N = 8)	Outpatient, vaccinated (N = 16)	p value
DPSO					0.50^a^
Median (Q1, Q3)	16.0 (14.5, 19.5)	17.5 (14.8, 20.0)	18.5 (14.8, 20.0)	15.0 (15.0, 16.0)	
Min - Max	14.0 - 21.0	14.0 - 20.0	14.0 - 21.0	14.0 - 20.0	
Days post first positive					0.23^a^
Median (Q1, Q3)	11.0 (8.0, 16.0)	13.5 (13.0, 16.2)	18.0 (11.5, 21.2)	15.0 (10.0, 18.2)	
Min - Max	2.0 - 20.0	13.0 - 23.0	5.0 - 25.0	9.0 - 346.0	
Age					<0.01^a^
Median (Q1, Q3)	48.0 (37.5, 61.5)	24.0 (22.0, 27.0)	61.0 (50.5, 71.8)	44.5 (32.8, 65.2)	
Min - Max	25.0 - 72.0	19.0 - 33.0	43.0 - 87.0	20.0 - 76.0	
Sex	8 (72.7%)	2 (50.0%)	6 (75.0%)	11 (68.8%)	0.83^b^
Race					<0.01^b^
White	10 (90.9%)	0 (0.0%)	7 (87.5%)	12 (75.0%)	
Black	1 (9.1%)	1 (25.0%)	1 (12.5%)	2 (12.5%)	
Asian or Pacific Islander	0 (0.0%)	0 (0.0%)	0 (0.0%)	1 (6.2%)	
Multiple	0 (0.0%)	2 (50.0%)	0 (0.0%)	0 (0.0%)	
Other	0 (0.0%)	1 (25.0%)	0 (0.0%)	1 (6.2%)	
Ethnicity: Non-Hispanic	9 (81.8%)	2 (50.0%)	8 (100.0%)	11 (68.8%)	0.19^b^
CCI					0.07^a^
Median (Q1, Q3)	1.0 (0.0, 2.5)	0.0 (0.0, 0.2)	3.5 (1.0, 6.0)	0.5 (0.0, 3.0)	
Min - Max	0.0 - 8.0	0.0 - 1.0	0.0 - 11.0	0.0 - 5.0	

a Kruskal-Wallis rank sum test.

b Pearson’s Chi-squared test.

CCI, Charlson Comorbidity Index; DPSO, days post symptom onset.

**Figure 2:**
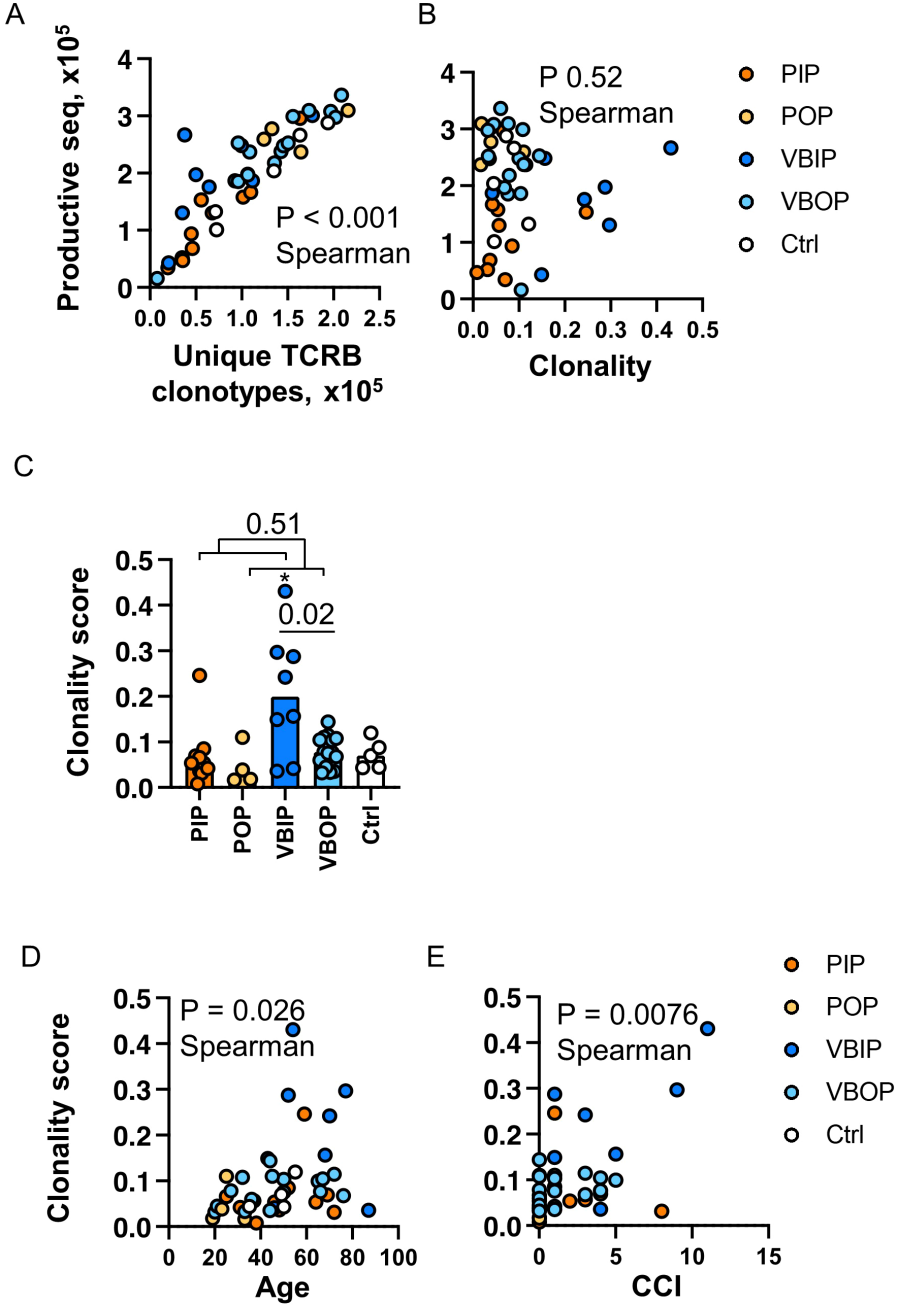
Clonality of the TCRB repertoire is associated with increasing age and comorbidities. **(A)** Correlation of sequencing depth and identification of clonotypes. P value indicates the significance by Spearman correlation. **(B)** Correlation of sequencing depth and clonality score. **(C)** Comparison of clonality score of TCR repertoires between study groups. P values indicate the significance by Mann-Whitney test. Asterisk indicates p <0.05 significance by Kruskal-Wallis rank sum test. Bars represent the median value in each group. **(D)** Correlation of TCR clonality and ages of participants. **(E)** Correlation of TCR clonality and Charlson Comorbidity Index (CCI) of participants. P value indicates the significance by Spearman correlation. PIP, primary infection inpatients; POP, primary infection outpatients; VBIP, vaccine breakthrough infection inpatients; VBOP, vaccine breakthrough infection outpatients.

A range of 15,889 to 336,571 total productive TCRB sequences were acquired per participant. The number of unique clonotypes ranged from 7,709 to 215,696 per participant, and was linearly correlated with the number of productive TCRB sequenced (**Figure 2A**), indicating that the sequencing depth was correlated with the number of TCR clones identified. Therefore, we next analyzed the clonality which is inversely related to repertoire diversity and has been used to characterize immune fitness (42, 43). The clonality score was calculated as one minus the normalized Shannon entropy, resulting in values ranging from 0 (high diversity) to 1 (monoclonality) (44–46). A higher score reflected higher clonality, thus lower diversity of the TCRB repertoire. The clonality score was not impacted by sequencing depth, therefore we used it to compare all participants (**Figure 2B**). The distribution of clonotypes by count among the study participants was not impacted by the variability in sequencing yields or study groups (**Supplementary Figure S1**). 78 - 92% of the total TCRB clonotypes had only one count regardless of the sequencing depth. Upon comparing the clonality score between clinical groups, we found that the VBIP group had the highest clonality score (**Figure 2C**). Analysis of the clonality score across participants demonstrated a positive correlation with age (**Figure 2D**). Inclusion of comorbidities in the correlation analysis using the CCI (47, 48) showed the strongest correlation with clonality (**Figure 1E**) among all participants. This was consistent with our finding that VBIP had the highest clonality score and the highest median age and CCI (**Figure 2C** and **Table 1**).

These data demonstrate that the majority of unique TCR clones measured in the peripheral blood have low count numbers. The depth of sequencing therefore increases the absolute number of unique clones but the distribution of clonal frequency did not differ by sequencing depth. Increasing age and comorbidities have been demonstrated as risk factors of COVID - 19 severity and the association with TCR diversity may provide additional insight into COVID - 19 severity.

### 2.2 Optimizing database analysis to enrich for SARS-CoV-2-specific CD8 TCRs

In order to maximize our analysis of SARS-CoV-2-specific epitopes, we chose to combine pre-existing databases and publications that defined SARS-CoV-2-specific CD8 TCR sequences. We first compared the TCRB sequences from our study participants with the ImmuneCODE MHC-I database (Release002, Adaptive Biotechnologies) that contains ~150,000 SARS-CoV-2-specific CD8 TCRB sequences and their corresponding epitopes (38). A CD8 TCRB sequence from our study participants that matched the CDR3β sequence, V gene, and J gene of a recorded sequence in the ImmuneCODE database was considered specific to the corresponding epitope. The frequencies of SARS-CoV-2-specific CD8 TCRB were calculated using the ImmuneSEQ analyzer (Adaptive Biotechnologies) (38, 39) and the LymphoSeq package (46) in R by viral antigens. The frequencies of SARS-CoV-2-specific CD8 TCRB using these two methods were comparable among viral spike (S), nucleoprotein (N), and open reading frame (ORF)1ab viral antigens (**Supplementary Figure S2A**). However, these analyses also revealed that pre-pandemic healthy controls had SARS-CoV-2-specific CD8 TCRB sequences detected at similar frequencies as SARS-CoV-2 positive study participants during peak T cell response (**Supplementary Figure S2A**). These findings indicated that the ImmuneCODE database included TCRB sequences that were non-specific for SARS-CoV-2 or cross-reactive with other non-SARS-CoV-2 antigens, limiting the ability to track SARS-CoV-2-specific TCRs.

**Figure 3:**
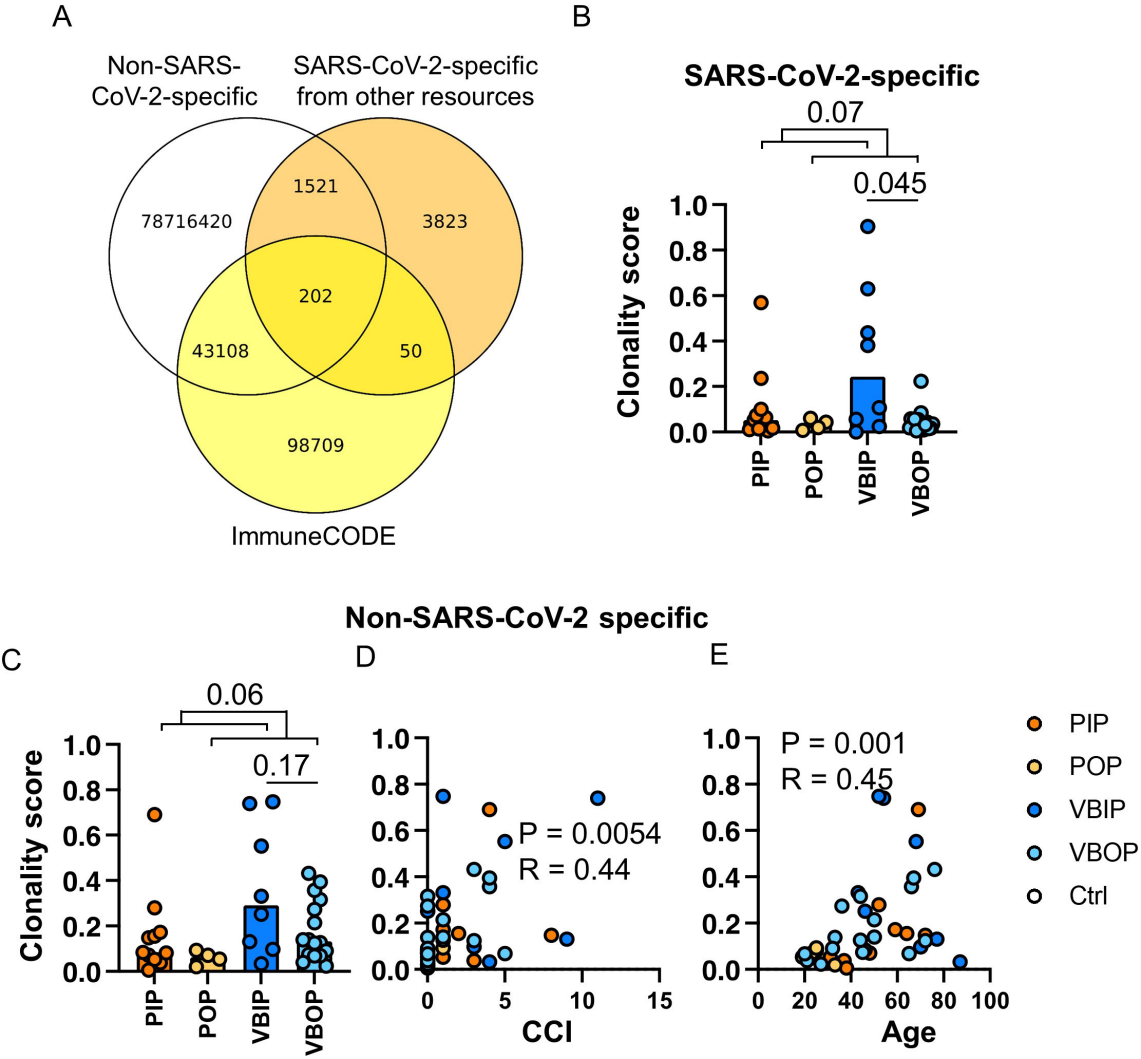
The clonality of SARS-CoV-2-specific CD8 TCRB among study groups. **(A)** Schematic illustration of the approach to identifying SARS-CoV-2-specific CD8 TCRBs using the pre-pandemic sequencing data (white), ImmuneCODE database (yellow) and other published SARS-CoV-2-specific CD8 TCRB (orange). The pool of 3,832, 50, and 98,709 sequences were used for downstream analyses as SARS-CoV-2-specific. **(B)** The clonality of SARS-CoV-2-specific CD8 TCRB among study groups. P values show significance based on Mann-Whitney test. **(C)** The clonality of CD8 TCRB not specific for SARS-CoV-2 as determined by the VDJ database and compared between clinical groups. **(D, E)** The correlation of the clonality of non-SARS-CoV-2 CD8 TCRB found in the VDJ database and CCI **(D)** or age **(E)**. The P values indicate the significance by Spearman correlation.

In order to increase SARS-CoV-2 specificity, we compiled a meta-database incorporating the ImmuneCODE database and other published repertoires (**Figure 3A**, **Table 2**). We enhanced the comprehensiveness of our analysis by assembling over 78 million pre-pandemic CD8 TCRB sequences from our healthy controls and TCR repertoires published before January of 2020, as well as over 5,300 SARS-CoV-2-specific CD8 TCRB sequences from the VDJdb, and additional sequences from published tetramer-sorted SARS-CoV-2-specific CD8 T cells (**Table 2**). While cross-reactive T cells play a role in the SARS-CoV-2 response (9, 17), given the high number of proposed SARS-CoV-2-specific epitopes found in our pre-pandemic controls, we chose to focus our analysis on TCRs with the highest confidence for SARS-CoV-2 specificity. To maximize SARS-CoV-2 specificity, we excluded CD8 TCRBs sequenced prior to 2020, considering them to be either cross-reactive or non-specific. As a result, 102,582 CD8 TCRB sequences were considered SARS-CoV-2-specific and used in the CD8 TCRB sequence database to analyze the TCRs sequenced in this study (**Figure 3A**).

**Table 2:** TCR sequence data gathered from databases or other studies, used in construction of TCR meta-database.

Dataset type	Database source/Study Title	Ref.
Pre-pandemic	*Clonally expanded CD8 T cells patrol Alzheimer’s cerebrospinal fluid*	(108)
Pre-pandemic	*Peripheral clonal expansion of T lymphocytes associates with tumor infiltration and response to cancer immunotherapy*	(109)
Pre-pandemic	*Single T Cell Sequencing Demonstrates the Functional Role of αβ TCR Pairing in Cell Lineage and Antigen Specificity*	(110)
Pre-pandemic	10x Genomics, CD8+ T cells of Healthy Donor 1	(111)
Pre-pandemic	10x Genomics, CD8+ T cells of Healthy Donor 2	(112)
Pre-pandemic	10x Genomics, CD8+ T cells of Healthy Donor 3	(113)
Pre-pandemic	10x Genomics, CD8+ T cells of Healthy Donor 4	(114)
Pre-pandemic	*Single-cell Map of Diverse Immune Phenotypes in the Breast Tumor Microenvironment - 5’ RNA sequencing and TCR sequencing*	(115)
Pre-pandemic	*CMV reactivation drives post-transplant T cell reconstitution and results in defects in the underlying TCRβ repertoire.* Database accessed from Adaptive Biotechnologies.	(116)
Pre-pandemic	*PD-1 blockade induces responses by inhibiting adaptive immune resistance.* Database accessed from Adaptive Biotechnologies.	(117)
Pre-pandemic	*Estimating the ratio of CD4+ to CD8+ T cells using high-throughput sequence data.* Database accessed from Adaptive Biotechnologies.	(118)
Pre-pandemic	*Long-term maintenance of human naive T cells through in situ homeostasis in lymphoid tissue sites.* Database accessed from Adaptive Biotechnologies.	(119)
Pre-pandemic	*Selective Expansion of High Functional Avidity Memory CD8 T Cell Clonotypes During Hepatitis C Virus Reinfection and Clearance.* Database accessed from Adaptive Biotechnologies.	(120)
Pre-pandemic	*Broad TCR repertoire and diverse structural solutions for recognition of an immunodominant CD8+ T cell epitope.* Database accessed from Adaptive Biotechnologies.	(121)
Pre-pandemic	*High throughput T cell receptor sequencing identifies clonally expanded CD8+ T cell populations in Alopecia Areata.* Database accessed from Adaptive Biotechnologies.	(122)
Pre-pandemic	*CD4+ and CD8+ autoreactive T cells in narcolepsy patients target self-antigens of hypocretin-producing neurons.* Database accessed from Adaptive Biotechnologies.	(123)
Pre-pandemic	*Multifactorial Heterogeneity of Virus-specific T Cells and Association with the Progression of Human Chronic Hepatitis B Infection.* Database accessed from Adaptive Biotechnologies.	(124)
Pre-pandemic	*Immunosequencing identifies signatures of cytomegalovirus exposure history and HLA-mediated effects on the T-cell repertoire.* Database accessed from Adaptive Biotechnologies.	(125)
SARS-CoV-2-specific by tetramer or AIM^1^	*Perturbations of the T-cell receptor repertoire in response to SARS-CoV-2 in immunocompetent and immunocompromised individuals*	(95)
SARS-CoV-2-specific by tetramer or AIM^1^	*Allelic variation in class I HLA determines CD8+ T cell repertoire shape and cross-reactive memory responses to SARS-CoV-2*	(126)
SARS-CoV-2-specific by tetramer or AIM^1^	SARS-CoV-2-specific CD8+ T-cell responses and TCR signatures in the context of a prominent HLA-A*24:02 allomorph	(127)
SARS-CoV-2-specific by tetramer or AIM^1^	SARS-CoV-2-reactive T-cell receptors isolated from convalescent COVID - 19 patients confer potent T-cell effector function	(128)
SARS-CoV-2-specific by tetramer or AIM^1^	*CD8+ T cells* sp*ecific for an immunodominant SARS-CoV-2 nucleocapsid epitope cross-react with selective seasonal coronaviruses*	(129)
SARS-CoV-2-specific by tetramer or AIM^1^	*SARS-CoV-2 antigen exposure history shapes phenotypes and* sp*ecificity of memory CD8+ T cells*	(130)
Databases	VDJdb+C6C2:C2C2:C27	(41)

^1^Activation induced marker.

Using this CD8 TCRB meta-database, the clonality scores of CD8 TCRB predicted to recognize epitopes from SARS-CoV-2 proteins were compared among study groups. Following the same trend as the total TCR clonality, total SARS-CoV-2-specific CD8 TCRB VBIP group had higher clonality scores compared to VBOP group (**Figure 3B**). Comparing inpatient to outpatient clinical groups revealed a trend towards higher clonality scores among inpatients that did not meet statistical significance. The clonality of SARS-CoV-2-specific CD8 TCRB sequences was weakly correlated with age but not CCI (**Supplementary Figure S2B**).

We further analyzed the clonality score of CD8 TCRB sequences identified to be specific for non-SARS-CoV-2 antigens as defined in the VDJdb to characterize the pattern of diversity of other identified CD8 TCRs. This analysis included but was not limited to TCRs responsive to cancers, diabetes, and chronic infections such as cytomegalovirus (CMV) and Epstein–Barr virus (EBV), which were identified by sequencing tetramer-sorted CD8 T cells. The identified non-SARS-CoV-2-specific CD8 TCRB also showed a non-significant trend in the clonality scores between clinical groups (**Figure 3C**). The clonality scores of these non-SARS-CoV-2 CD8 TCRB sequences were positively correlated with CCI (**Figure 3D**) and age (**Figure 3E**).

For this analysis, we focused on CD8 TCRs given the relative strength of the ImmuneCODE database for MHC-I over MHC-II (>140,00 TCRs compared to 6,360 TCRs, respectively). When we used the same approach with CD4 TCRs, incorporating other published CD4 repertoires (**Table 2**) and removing TCRB sequences that had been identified pre-pandemic, we found a non-significant trend towards higher SARS-CoV-2-specific clonality in participants who experienced severe compared to mild disease (**Supplementary Figures S3A, B**). The majority of SARS-CoV-2-specific TCRS were specific to non-S epitopes, and vaccinated participants did not have higher frequencies of S-specific TCRs (**Supplementary Figures S3C, D**).

Together, these results demonstrate that increasing the reference sequencing data for both antigen-specific and non-specific TCRs improves the bioinformatic potential to identify TCR specificity. The non-SARS-CoV-2-specific TCR dataset correlated with CCI indicating that this aspect of the database enriched for TCRs associated with other chronic diseases.

### 2.3 The frequency of SARS-CoV-2 protein recognition by CD8 T cells differed by vaccination and clinical status

**Figure 4:**
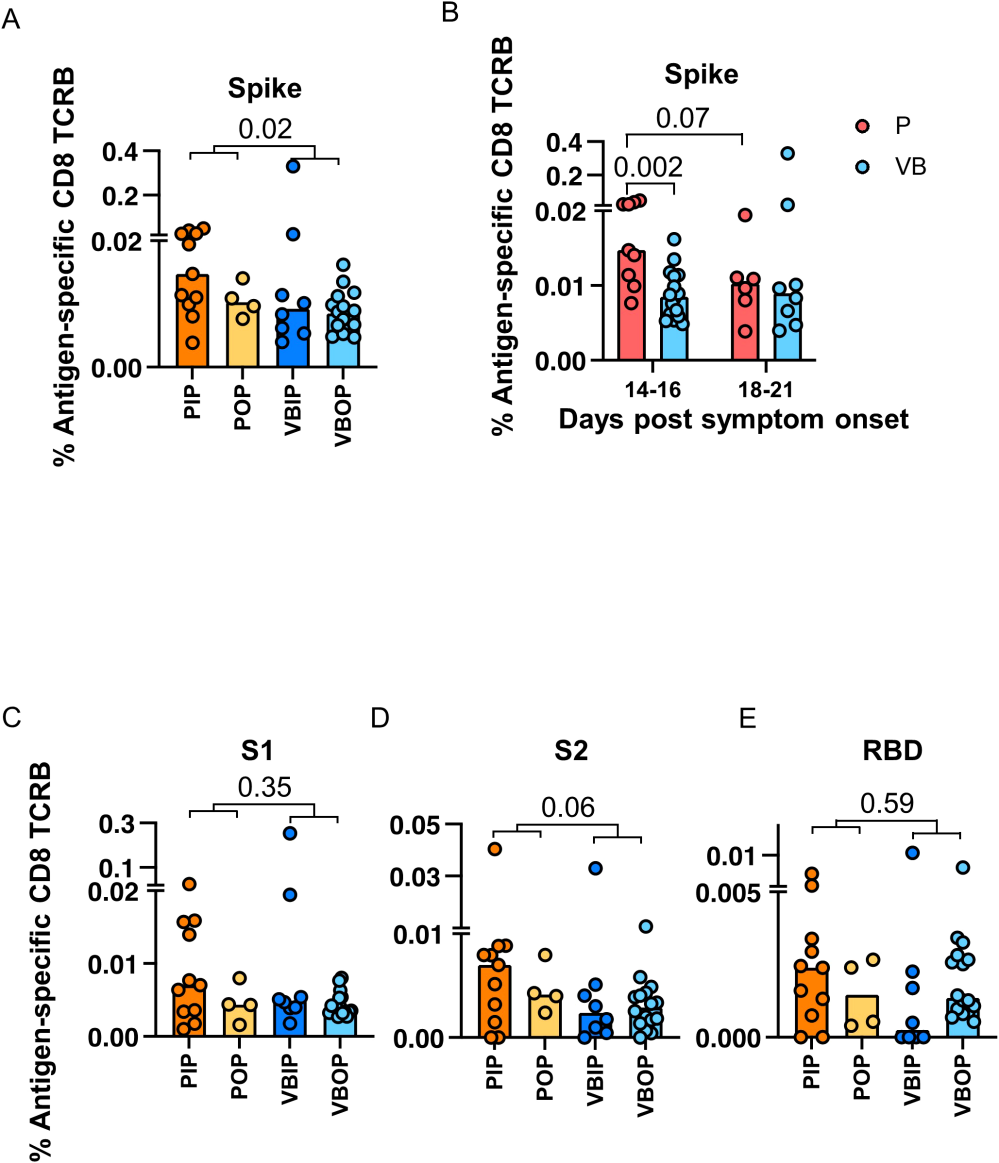
The frequencies of SARS-CoV-2 antigen-specific CD8 TCRB among study groups. **(A)** Frequency of Spike-specific CD8 TCRB among study groups. P values indicate significance by Mann-Whitney test. **(B)** Frequencies of Spike-specific CD8 TCRB measured in primary (red) and vaccine breakthrough (blue) participants by 14 to 16 and 18 to 21 days post symptom onset. P values indicate the significance by Mann-Whitney test. **(C-E)** Frequency of S1 **(C)**, S2 **(D)** and RBD **(E)**-specific CD8 TCRB among study groups.

Upon identifying CD8 TCR repertoire patterns that were associated with COVID - 19 severity and vaccination, we next asked if specific TCR-epitope recognition differed by vaccination status. The frequency of SARS-CoV-2 antigen-specific CD8 TCRB sequences was calculated as the percentage of accumulated specific CD8 TCRs within each viral protein out of total productive TCR sequences. Interestingly, unvaccinated participants had higher frequencies of S-specific CD8 TCRB sequences compared to vaccine breakthrough participants (**Figure 4A**).

In order to understand the higher frequency of S-specific TCRs in participants with primary infection, we evaluated the kinetics of the S-specific TCR response. The frequency of S-specific CD8 TCRB at 14 – 16 days post symptoms onset (DPSO) in participants with primary infection was higher than that in participants with vaccine breakthrough infection, but were comparable 18 – 21 DPSO (**Figure 4B**). By contrast, the CD4 TCRB showed no difference (**Supplementary Figure S3E**). These findings suggested that vaccine-induced S-specific memory CD8 cells had possibly expanded prior to 14 DPSO or differed in their pattern of expansion associated with viral load or other host immune factors. When sub-dividing CD8 TCRs with predicted specificity to S1 or S2, we saw no difference with either subunit, although there was a trend towards higher frequencies of TCRB specific for S2 in the primary infection group (**Figures 4C, D**). Although not significant, the frequencies of RBD-specific CD8 TCRB sequences in the VBIP group trended towards the lowest among study groups (**Figure 4E**), suggesting that some VBIP participants may have impaired ability to develop T cell responses against novel epitopes provided by mRNA vaccination followed by breakthrough infection.

**Figure 5:**
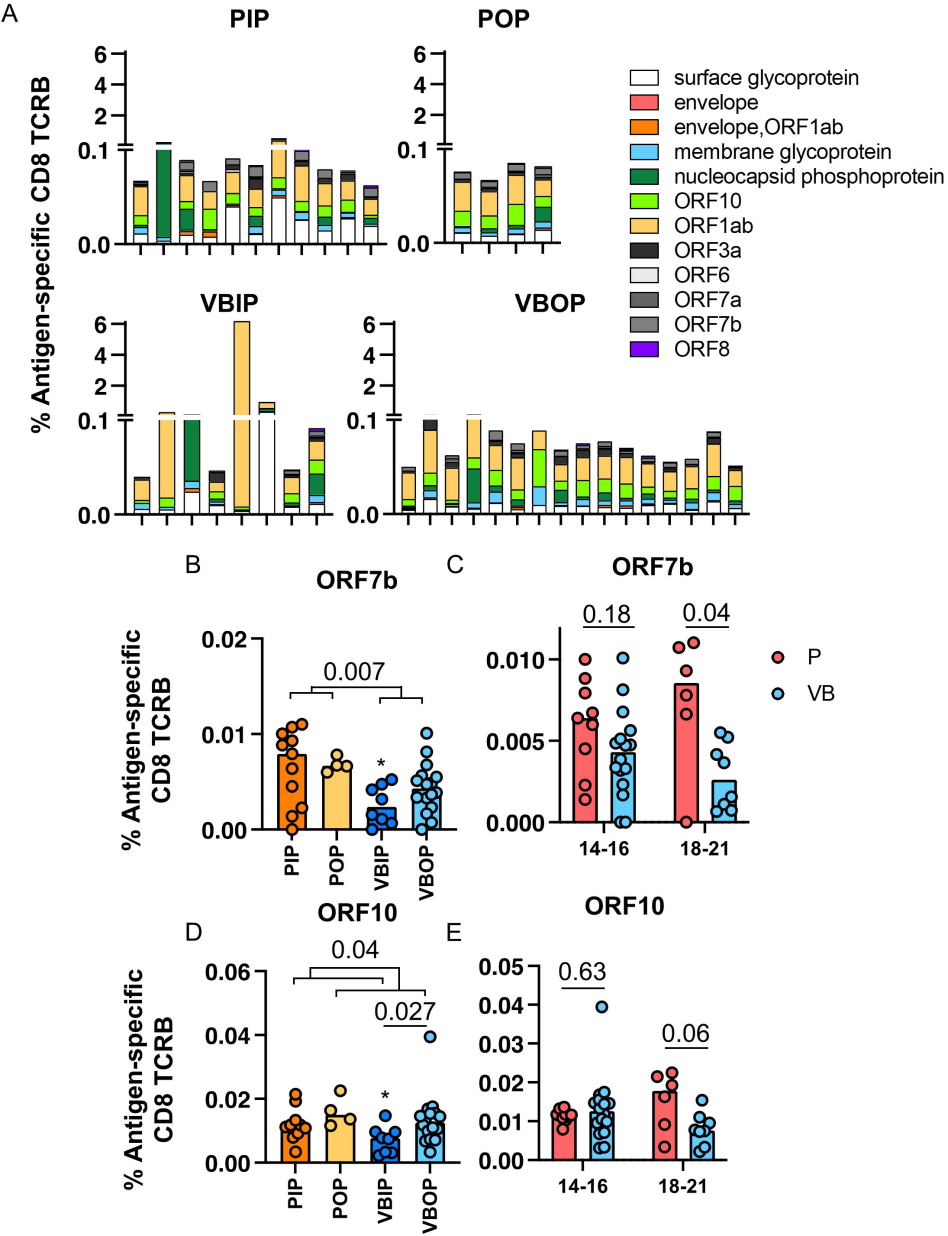
Frequencies of SARS-CoV-2-specific CD8 TCRB by antigens. **(A)** Frequencies of SARS-CoV-2-specific CD8 TCRB by viral antigens in each participant among the study groups. **(B, C)** Frequency of ORF7b **(B)** ORF10 **(C)** -specific CD8 TCRB among study groups. P values indicate significance by Mann-Whitney test. **(D, E)** Frequencies of ORF7b **(D)** and ORF10 **(E)**-specific CD8 TCRB measured in primary (red) and vaccine breakthrough (blue) participants between DPSO grouped as 14 to 16 and 18 to 21.

Amongst all SARS-CoV-2 proteins, spike glycoprotein did not dominate the TCR response. Upon comparison of the distribution of viral antigens recognized by TCRBs, ORF1ab was dominantly recognized regardless of history of vaccination (**Figure 5A**). ORF1ab-specific TCRs on average comprised 37% of all the identified SARS-CoV-2-specific TCRB sequences in our participants. Comparing non-spike proteins between groups, we found that participants experiencing primary infection exhibited higher ORF7b-specific CD8 TCRB (**Figure 5B**). ORF7b is a small 43 amino acid membrane-associated protein that has been shown to impair innate immunity by interfering with the RIG-I-like receptor pathway to limit type I interferon (IFN-I) and tumor necrosis factor-α (49–53). We compared the frequency of ORF7b-specific CD8 TCRB at 14 – 16 and 18 – 21 DPSO. Unlike S-specific CD8 TCRB, the ORF7b-specific CD8 TCRB exhibited a lower frequency at 18 – 21 DPSO in the participants experiencing vaccine breakthrough infection, but was comparable at the earlier time point (**Figure 5C**) suggesting a distinct pattern of expansion and contraction of T cells specific for this protein. Between inpatients and outpatients, ORF10-specific CD8 TCRB sequences were higher in the outpatients and the VBIP had the lowest frequency of ORF10 epitope recognition (**Figure 5D**). ORF10 is a highly conserved protein that has been proposed to limit IFN stimulated genes and IFN-production (54–57).The kinetics of the measured ORF10-specific CD8 TCRs showed lower frequency at the later, 18 – 21 DPSO time point, but not the earlier time point in the participants experiencing vaccine breakthrough infection, similar to ORF7b (**Figure 5E**). These findings suggest that kinetics of the S-specific TCRs are more consistent with a memory response (6) in VB participants. T cells responding to ORF7b and ORF10 were lower at the later time point in vaccinated participants, possibly reflecting better virus control (**Figures 5C, E**).

These data indicate that the T cell response across the SARS-CoV-2 proteome was broad and not spike-dominant, although this may be influenced by kinetics of the response and the timing of sampling in this study. Additionally, CD8 T cells recognizing epitopes in ORF7b and ORF10 may play a role in pathogenesis (51, 52, 54). T cells recognizing epitopes within these proteins may influence disease outcomes.

### 2.4 Hierarchy of SARS-CoV-2 peptide recognition by CD8 T cells differed based on disease severity

**Figure 6:**
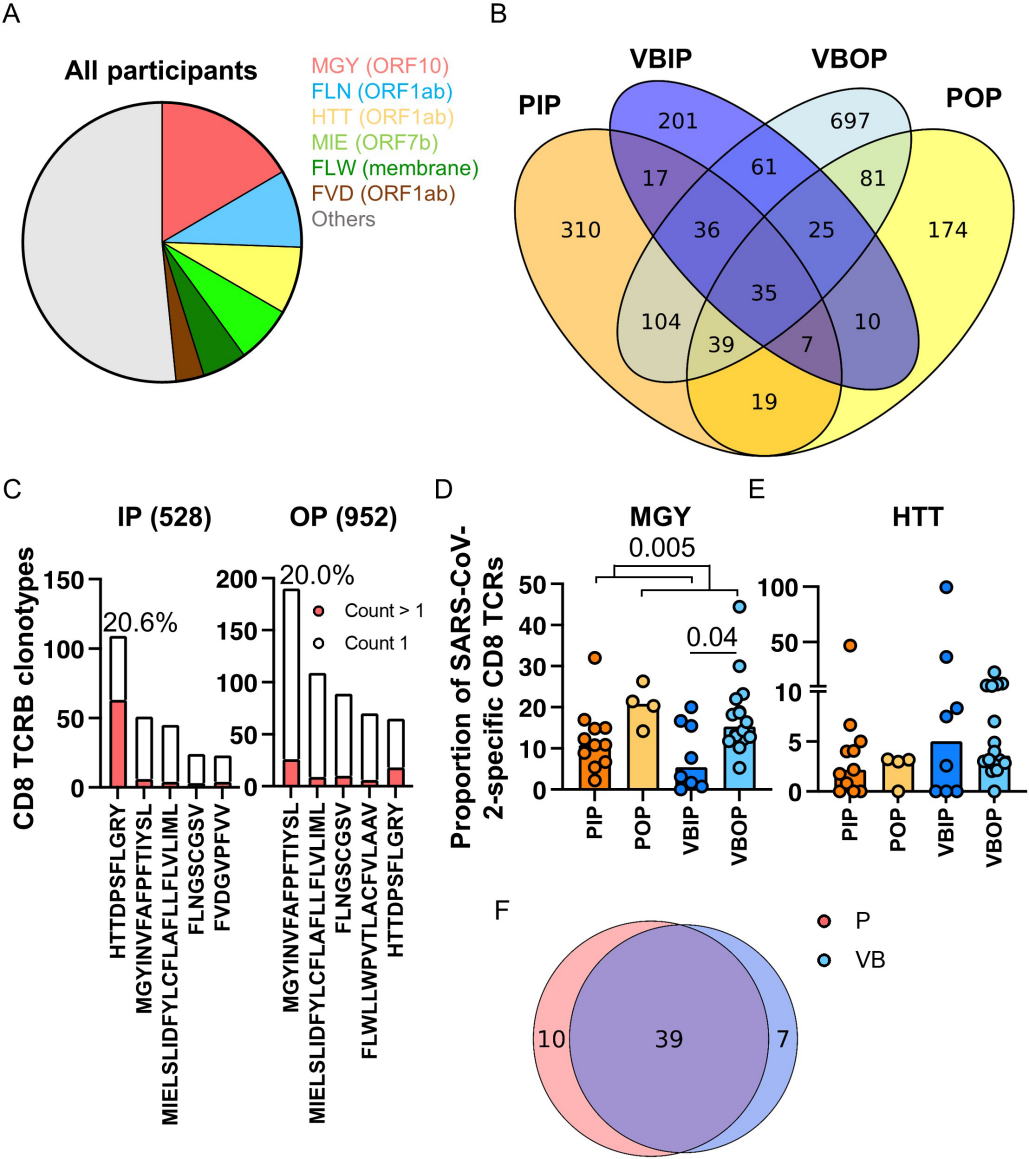
Pattern of peptide-specific CD8 TCRB and common epitopes recognized by clinical disease and vaccination status. **(A)** SARS-CoV-2 peptide distribution in all participants. The slices represent the top 5 most commonly recognized peptides. **(B)** Venn diagram of SARS-CoV-2-specific CD8 TCRB sequences among all study groups. **(C)** The ranks of the top 5 common peptides recognized by the TCRs found exclusively in the inpatient (528) or outpatient (952) groups. Red bars represent TCRs with at least 2 counts within a participant. **(D, E)** Proportion of SARS-CoV-2-specific CD8 TCRB recognizing MGY **(D)** or HTT **(E)** peptide among study groups. The P values indicate the significance by Mann-Whitney test. **(F)** Venn diagram of peptides in Spike recognized by primary (red) and vaccine breakthrough (blue) patients.

Upon determining that the frequency of TCRB recognizing SARS-CoV-2 proteins differed by illness severity and vaccination status, we next asked if specific epitopes were differentially recognized between clinical study groups. Using our meta-database approach to define the epitopes corresponding to the SARS-CoV-2-specific CD8 TCRB sequences, we characterized the immunodominant epitopes (**Figure 6A**) from peptide pools from databases or studies that were defined as restricted to MHC-I (**Table 1**). Interestingly, 16.6% of the sequenced CDR3 targeted peptide MGYINVFAFPFTIYSL (MGY_ORF10_) (**Figure 6A**). MGY_ORF10_ is a 16 amino acid peptide in ORF10 assembled by 7 peptides used to identify SARS-CoV-2-specific CD8 T cells by Multiplexed assay for Identification of T cell Receptor Antigen Specificity (MIRA) (Adaptive Biotechnologies) (38). Although the peptide pool for MGY_ORF10_ covered only 42.1% of ORF10, this region of the protein was highly recognized in our participants. Together with the MGY peptide, 48.4% of SARS-CoV-2-specific CD8 TCRB clonotypes recognized six peptides distributed in ORF1ab, ORF7b, ORF10, and membrane (**Figure 6A**). Peptide pools from ORF10, membrane, ORF7a and Spike represented the majority of identified CD4 epitopes (**Supplementary Figure S3C**).

Across clinical groups a range of 35.3%-55.4% of SARS-CoV-2-specific CD8 clonotypes were shared (**Figure 6B**). We hypothesized that SARS-CoV-2 CD8 peptide recognition exclusively in outpatients were associated with improved clinical outcomes. 528 clonotypes were identified exclusively from participants with severe COVID - 19 and 952 clonotypes were exclusive to those with mild disease (**Figures 6B, C**). The clonotypes distinct to inpatients or outpatients recognized common viral peptides with differing hierarchies (**Figure 6C**). Outpatients dominantly (20.0%) recognized MGY from ORF10 while inpatients dominantly (20.6%) recognized HTTDPSFLGRY (HTT_ORF1ab_) from ORF1ab (**Figure 6C**), although there was significant overlap in common peptides between the two groups, which suggests either the importance of these peptides in the overall SARS-CoV-2 response, or differences in presentation. Most TCR clonotypes recognizing the top peptides had one count, except for the HTT_ORF1ab_ peptide in which 57.8% of the clonotypes had more than one count. In addition, sequences recognizing MGY_ORF10_ comprised a median of 17.3% of all SARS-CoV-2-specific CD8 TCRB sequences in outpatients, significantly higher than 10.8% in inpatients (**Figure 6D**). HTT_ORF1ab_ was the next most commonly recognized epitope and comprised a median of 3.2% of all SARS-CoV-2-specific CD8 TCRB sequences and no difference was measured among study groups (**Figure 6E**). However, TCR recognition of the HTT_ORF1ab_ peptide reflected 99.5% of the SARS-CoV-2-specific TCRs in one VBIP participant, highlighting variability in participant responses and the restricted repertoire exhibited by participants with severe disease.

As S was included in the mRNA vaccines, we next compared the TCRB S epitope recognition between vaccinated and unvaccinated participants. Unlike the other dominant epitopes, the most commonly recognized epitope in S represented 1.14% of the clonotypes, while the total S-specific response comprised 13.8% of the clonotypes. No significant difference in S epitope distribution was observed between unvaccinated and vaccine breakthrough participants (**Figure 6F**).

Since CD8 epitope presentation is influenced by HLA-I alleles, peptides containing epitopes presented by common HLA-A, -B, and -C alleles were analyzed. We used transcriptomic data to identify HLA-I alleles in this sub-cohort (**Supplementary Table S1**). Analysis of TCRB frequencies was performed for peptides presented by common alleles that were represented in 4 or more participants (**Supplementary Figures S4A-C**). Interestingly, peptide MGY_ORF10_ was found in 15 of 16 listed common HLA-I, suggesting that MGY_ORF10_ contains multiple epitopes and can be presented by a variety of HLA-I alleles.

In summary, these data suggest that the peptide MGY within ORF10 contains commonly presented epitopes and is highly represented among T cells recognizing SARS-CoV-2 in the participants with mild disease. Participants with severe disease alternatively recognized the HTT peptide from ORF1ab, which may indicate usage of less specific T cells as ORF1ab has been shown to have peptides cross-reactive to endemic coronaviruses (58). Inclusion of proteins such as ORF10 into vaccine design may provide more opportunities for T cell epitope recognition, improving memory T cell breadth.

### 2.5 Major histocompatibility usage and TCRB pattern recognition of ORF10 MGY and ORF1ab HTT peptides

**Figure 7:**
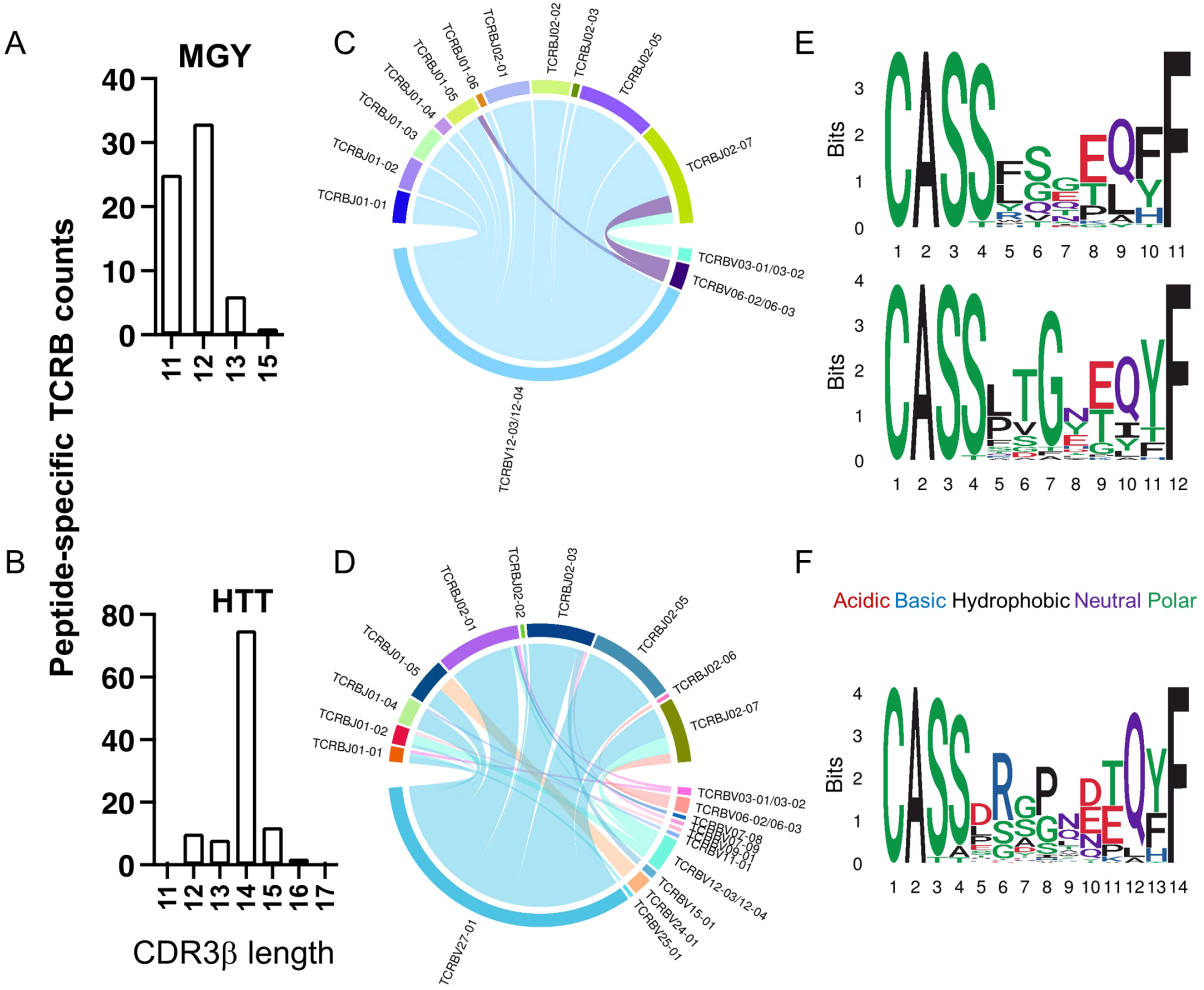
Characterization of CD8 TCRB recognizing peptides MGY_ORF10_ and HTT_ORF1ab_. **(A, B)** The distribution of the amino acid lengths in the CDR3β recognizing MGY_ORF10_ **(A)** and HTT_ORF1ab_ **(B)**. **(C, D)** V and J gene association among TCRB sequences recognizing MGY_ORF10_ **(C)** and HTT_ORF1ab_ **(D)**. **(E, F)** Web logo plots of consensus CDR3β amino acid sequences with 11 (top) or 12 (bottom) amino acids that recognize MGY_ORF10_ **(E)** and 14 amino acids that recognize HTT_ORF1ab_ **(F)**.

Next, we sought to characterize the distinct CDR3β variable (CDR3B) sequences that recognized the most frequently recognized peptides between those with severe versus mild disease. The patterns within the CDR3B of the TCR that determine the recognition of the two peptides were predicted using GLIPH2 (59). The TCRs recognizing MGY_ORF10_ predominantly had 11 or 12 amino acids (**Figure 7A**), while those recognizing HTT_ORF1ab_ predominantly had 14 amino acids in the CDR3B region (**Figure 7B**). The MGY_ORF10_-recognizing TCRs with 11 or 12 amino acids in the CDR3B were associated with V12 - 03/04 gene, and a variety of J genes except for J02 - 04/06 (**Figure 7C**). On the other hand, the HTT_ORF1ab_-recognizing TCRs with 14 amino acids in the CDR3B were dominantly associated with V27 - 01, and a variety of J genes except for J01 - 03/06 and J02 - 04 (**Figure 7D**). Among the MGY_ORF10_-recognizing TCRs with 11 amino acids in the CDR3B, those with amino acids QET at positions 6 – 8 were predicted as the top cluster of TCRs, followed by GQP at the same positions (**Figure 7E**, top). Among the MGY_ORF10_-recognizing TCRs with 12 amino acids in the CDR3B, those with PTG at positions 5 – 7 were predicted as the top cluster, followed by GET at positions 7 - 9, but glycine (G) at position 7 was the most common among these sequences (**Figure 7E**, bottom). For the HTT_ORF1ab_-recognizing TCRs with 14 amino acids as the dominant pattern, those with RGP at positions 6 – 8 were predicted as the top cluster (**Figure 7F**). Using these criteria including the length, consistent V gene, and motifs determined in GLIPH2, additional TCR sequences not previously identified in the database could be predicted. A median of 5.8 times (Q1-Q3 = 4.2 to 9.5) as many TCRs could be identified with the corresponding motif.

Together, we identified 6 epitope regions in four SARS-CoV-2 viral proteins most frequently recognized in our study cohort. Our data suggests that TCRs recognizing two of these epitope regions predominantly use distinct V and J genes however the potential to reassort with alternate V and J genes allowing for broader population wide TCR usage. Our findings explore the identification of key amino acids that may confer TCR affinity for viral epitopes. Continued evaluation of TCR motifs and key amino acid binding sites may facilitate the identification of TCRs that provide more cross-reactive versus specific epitope recognition.

## 3 Discussion

T cell immunity is important in controlling viral clearance upon primary and breakthrough SARS-CoV-2 infections (60). Characterization of T cells recognizing vaccine and viral epitopes is critical to understanding host immunity against SARS-CoV-2 infection and optimizing vaccine approaches. Vaccination has significantly reduced the frequency and severity of COVID - 19, and in the United States this primarily reflects the efficacy of the mRNA vaccines (61–64). While antibody correlates of protection have been identified, the quality and specificity of T cell responses associated with vaccine efficacy and reduction in disease severity remain unclear. In this study, we compared the TCR repertoires of individuals who experienced primary infection or infection following mRNA vaccination and had either severe or mild disease. We demonstrated that highly clonal total TCR repertoires correlated with age and multiple comorbidities, which are key risk factors for severe COVID - 19, and reveal the importance of incorporating T cell metrics in disease risk algorithms. By developing a meta-database approach enriching SARS-CoV-2-specific TCRs, we determined that total SARS-CoV-2-specific CD8 TCRs were higher in vaccine breakthrough inpatients compared to vaccine breakthrough outpatients. Furthermore, response to the SARS-CoV-2 proteome was broad, even in vaccinated participants. The S-specific response was slightly lower at the peak of the T cell response in vaccinated participants compared to primary infection, but the frequency was not statistically significant between vaccinated and unvaccinated participants. Differences in the frequency of S-specific TCRs at different time points suggest that vaccination impacted the kinetics and epitope recognition of the S-specific T cell response. These changes in T cell frequency may reflect differences in viral load or apoptosis of highly activated T cells. We hypothesize that this is due to more rapid activation of T cells in vaccinated individuals and that these S-specific T cells may already be contracting at 14 – 16 DPSO. Indeed, another study has found that compared to unvaccinated individuals, vaccine associated CD8 T cells are activated earlier upon infection in vaccinated individuals (65). Interestingly, a reduced T cell response to ORF7b and ORF10 was associated with severe disease after vaccination suggesting that reduced T cell responses against these viral antigens may be important in disease pathogenesis.

T cell responses are host dependent, relying on MHC presentation of epitopes. Due to the diversity of MHC molecules in the population, this results in a broad presentation of SARS-CoV-2 epitopes and resulting T cell diversity. The complexity of population wide T cell responses has resulted in limitations in characterizing protective T cell responses against SARS-CoV-2. Activation induced marker (AIM) assays were frequently used to identify SARS-CoV-2-specific T cells during the pandemic (66–68). AIM assays depend upon T cell activation in response to peptides and are therefore limited by the peptide pools used to stimulate T cells, background marker expression and bystander T cell activation which may occur when stimulating PBMCs. Enzyme-linked immunosorbent spot (ELISpot) assay, Interferon gamma release assays (IGRA) or intracellular cytokine staining also only identify SARS-CoV-2-specific T cells based on the peptide pools under investigation (5, 8, 24). To create our meta-database, we chose to include epitopes with predicted SARS-CoV-2-specificity validated by a variety of methods, including both AIM and tetramer-sorted cells. Although these methods are not analogous, by incorporating them into our database we hoped to increase the breadth of SARS-CoV-2-specific and non-SARS-CoV-2-specific TCRB examined to cross-validate specificity of the TCR.

We identified up to 200,000 unique TCRs per participant. Advancing age has previously been associated with reduced diversity and increased clonality of the TCR repertoire, with variable patterns of clonality driven by CD8 T cells and memory T cell populations (69–72). More recently, studies have analyzed TCR repertoires as markers of aging and as possible indicators of disease susceptibility (69). Our study further supports these analyses by demonstrating that participants who experienced hospitalization with COVID - 19 after vaccination had the highest total TCR clonality scores. Additional studies have found less diverse TCR repertoires in individuals with COVID - 19 compared to healthy donors (73, 74). Limited T cell repertoire diversity was also associated with severe COVID - 19 by others (75, 76). Furthermore, comorbidities such as cancer and immunosuppression have been associated with reduced TCR repertoire diversity (72). Similarly, participants in our study with the highest number of chronic comorbidities had the most restricted TCR repertoires. Markers of biologic aging and predictions of risk for disease have increasingly become more attainable with artificial intelligence analysis of larger data sets (77).

Our study primarily describes patterns of TCR usage recognizing MHC Class I presented epitopes typically by CD8 T cells. Class I presented epitopes are more richly described than Class II presented epitopes, and reference data for CD4 T cell receptor sequencing is limited. Sparse CD4 data limits applications of TCR repertoire analysis to potential therapeutics and vaccine design, as CD4 T cells are important for viral control, correlate with long-term antibody response, and can recognize variants of concern (23, 78–80). Interestingly, both CD4 and CD8 TCR repertoires in our meta-database were directed primarily against non-S proteins, even in vaccinated individuals. S has been the most well characterized protein in regards to T cell responses (81). The ImmunoSeq assay peptide pools cover 66.3% of the S protein sequence compared to only 12.9% of the protein sequence of ORF1ab. Evaluation of non-S-specific T cell responses is necessary to define T cell correlates of protection and improve antigens for vaccine design. The breadth of the SARS-CoV-2 proteome response in both vaccinated and unvaccinated participants highlights the need for continued study of non-S proteins. Epitopes in the nonstructural, open reading frame, and membrane proteins have been selected for vaccine candidates with better response to viral variants (82–84). Early studies reported dominant CD4 and CD8 T cell responses to the S protein (68), but more recent studies indicated that the ORF1ab protein provides a significant number of epitopes recognized by T cells (85, 86), which we also observed in this study.

We found that vaccinated participants with severe disease had reduced frequencies of epitopes against ORF7b and ORF10. ORF10 is uniquely expressed in SARS-CoV-2, and it inhibits the innate immune response through mechanisms such as degrading mitochondrial antiviral signaling protein (MAVS) (54, 87). Although the mechanism is not fully understood, MAVS upregulates the expression of HLA-I as reported in Influenza A infection (88). It is possible that ORF10 in participants with severe COVID - 19 downregulates HLA-I-mediated antigen presentation, limiting epitope recognition by T cells.

Additionally, we analyzed the immunodominance hierarchy between clinical study groups. The SARS-CoV-2-specific CD8 TCRs from participants with severe COVID - 19 skewed towards recognition of HTT in the ORF1ab, but this pattern was not observed in the outpatients. HTT is reportedly recognized by CD8 T cells from individuals with HLA-A*01:01 (31, 85). This was also observed in our study, but presentation was not limited to only HLA-A*01:01. The frequencies of CD8 TCRs recognizing HTT_ORF1ab_ in the other haplotypes were in general much lower than those in HLA-A*01:01. Peptide MGY in ORF10 was widely recognized by TCRs in this study, suggesting that it contains multiple epitopes presented by a variety of haplotypes. Indeed, epitopes YINVFAFPF associated with HLA-A*02:01 and NVFAFPFTI associated with HLA-A*02:01/B*13:01 within the peptide MGY_ORF10_ have been reported (89). Although no convincing distribution of the HLA haplotypes was associated with the severity of COVID - 19 in this study, MGY from ORF10 was recognized more frequently in outpatients. Epitopes such as LSPRWYFYYL-HLA-B*07 are highly homologous with common cold coronavirus strains HKU1 and OC43 (85, 90). Therefore, TCRs recognizing LSPRWYFYYL in our data set largely overlapped with pre-pandemic TCRs and were excluded in the SARS-CoV-2-specific TCR database. Both MGY and HTT tend to be highly conserved across SARS-CoV-2 variants, and the ORF1ab and ORF10 proteins have been included in putative pan SARS-CoV-2 vaccines (56, 91).

We examined the TCRs identified in our data set against both SARS-CoV-2-specific and non-specific CD8 TCRB to increase specificity of SARS-CoV-2 TCRB predictions. By using TCR datasets published prior to the pandemic to identify non-specific TCRs, we likely reduce them from our dataset but also eliminated some cross-reactive TCRs that may contribute to immunopathogenesis (92–94). Our in-silico identification of epitope-specific TCRs is based upon *in vivo* and *in vitro* validation of the TCR specificity, however, we did not further validate epitope recognition from our participants. Our study design sought to build pipelines for in silico modeling to reduce the cost and complexity of analyzing T cell responses. While our findings support this as a useful tool, we also show that there are limitations to this approach including the potential loss of cross-reactive T cells and the inclusion of non-specific T cells.

HLA differences between participants in this study, and those that formed the basis of included databases may bias our findings. The breadth of our SARS-CoV-2-specific meta-database is limited to the study populations and the experiments that informed it. As these studies heavily featured participants from North America and Europe, TCRs may be biased towards HLA types that predominate in these populations, limiting broader applicability and potentially missing other important epitopes. We note that this study draws important conclusions based on a small sample size. Future studies involving a larger sample size may strengthen findings presented here and increasing the diversity of HLA haplotypes may better represent the broader population.

Variability in TCR sequencing depth impacts the number of clonotypes available for analysis creating an analytical challenge. We show that the number of productive TCR sequences directly correlated with the number of unique clonotypes in our assay. Our investigation of different approaches to TCRB analysis demonstrated that the measured total T cell clonality was not impacted by sequencing depth. In some studies, algorithms that remove all clones with one or two counts have been used to normalize the data set (95) and our findings show that this reduces the data set by 70 - 90%. This reduction of the analyzed data set likely results in different findings regarding the diversity, clonality and richness of the TCR repertoire. Many of the unique SARS-CoV-2-specific TCRB in our dataset were only represented by one count in the productive sequences. As different approaches to TCR sequencing are developed consideration for the approach to analysis will become increasingly important and need to be further explored. Use of RNA for TCR sequencing may have improved sensitivity for rare clonotypes. However, we elected to use genomic DNA for its improved stability to enable downstream sequencing, and to reduce potential bias introduced in both variable RNA copy numbers per cell (versus DNA, with 1 copy number corresponding to 1 cell) and during reverse transcription step (26). This approach required reliance on the TCRB, rather than paired α/β sequences, to predict epitope specificity. Crystalline structure analysis and computational approaches have shown that while TCRα chains interact with epitopes and can impact epitope specificity, the majority is driven by the TCRB chain (96, 97). Moreover, single cell TCR sequencing is required to obtain paired TCRα and TCRB chains. A paired read is obtained in 50 - 80% of T cells sequenced and typically single cell sequencing is performed on 1000 – 5000 T cells per participant (98, 99) resulting in limited sampling of the TCR repertoire. Substantially increasing the number of T cells sequenced becomes cost prohibitive with this technology. Therefore, sequencing of paired TCRα and TCRB chains remains less common than of TCRB alone (100), thus reliance on paired chains would limit use of pre-existing databases. Various models have been published to predict TCR and epitope recognition using sequences of paired α and β chains (101, 102). Models such as GLIPH2 (59) cluster TCRs based mainly on β chains, but the number of clusters as well as the sequences within each cluster are correlated with sequencing depth. Sequences similar to SARS-CoV-2-specific CD8 TCRs by clusters are more readily identified in participants with higher total sequence yield. On the other hand, we identified a few TCRs containing one amino acid difference in the CDR3B region compared to the predicted SARS-CoV-2-specific CD8 TCRs, which minimally contributed to the total counts of SARS-CoV-2-specific CD8 TCRs and may have variable binding *in vivo*. Therefore, future analysis of TCR binding and functional responses of SARS-CoV-2-specific CD8 T cells identified in this study would provide further insight.

Clonality metrics are one approach towards measuring the diversity and variability of T cell responses. Analysis of the predicted SARS-CoV-2-specific TCR repertoire allows practical input from across the studied SARS-CoV-2 proteome, enabling discovery of TCRs predictive of clinical outcomes. Further analysis of the phenotype and functionality of the T cells identified in our study is necessary to define impact on host immunity. Furthermore, the bioprocess of TCR-epitope recognition relies on antigen presentation by antigen-presenting cells. TCRs that are predicted to recognize a certain epitope based on the sequence and database may not do so if the epitope is not presented *in vivo*.

As virus evolution evades antibody neutralization, it becomes increasingly important to identify T cell epitopes that may provide more durable protection against SARS-CoV-2. S-specific T cells demonstrate cross-reactivity between emerging SARS-CoV-2 variants, and virus evolution has thus far not led to antigenic variation that facilitates complete T-cell escape (23). Furthermore, in silico analysis suggests that non-S protein recognition by T cells is conserved for newer strain variants such as BA.2.68 (25).

In summary, our study finds that restriction of both total and SARS-CoV-2-specific TCR repertoires is associated with COVID - 19 severity. Severe disease is also associated with reduced frequency of ORF7b and ORF10 epitope recognition and dominance of epitope recognition in ORF1ab. These findings highlight the potential to identify T cell patterns and targets associated with aging and comorbidities which are risk factors for severe respiratory disease. Further evaluation of these patterns and the development of comprehensive TCR databases to better define epitope recognition patterns may enhance vaccine design for high risk populations.

## 4 Materials and methods

### 4.1 Study participants

The EPICC study enrolled participants tested for or who had a high-risk exposure to COVID - 19, or were vaccinated against SARS-CoV-2 infection from March 2020 to May 2022, as reported previously (8, 103). Participants were enrolled at military treatment facilities (MTFs) located in the United States. Demographic and clinical data were collected at enrollment and specified follow-up time points. Infections with SARS-CoV-2 and vaccination history was reported by the participants and extracted from the participant’s medical record. All vaccinated participants in this sub-analysis had received either the BNT162b2 mRNA or mRNA-1273 COVID - 19 vaccine. Biospecimens for this study were included only if the participants were negative by PCR for other co-infecting viruses. Participants included in the primary infection study group were unvaccinated at the time of their first SARS-CoV-2 infection, while participants included in the vaccine breakthrough study group had a PCR confirmed SARS-CoV-2 infection at least 14 days after they received their second vaccine dose. The protocol was approved by the Uniformed Services University’s Institutional Review Board (IDCRP - 085), and all participants or their legally authorized representative provided informed consent to participate. Control participants (median age 49.5 years, range 35 - 55) were enrolled in a healthy donor protocol (R075QO) at the National Institute of Health and their peripheral blood biospecimens were collected prior to the pandemic in 2019.

### 4.2 PBMC isolation

PBMCs were isolated from peripheral whole blood collected in acid citrate dextrose (ACD) tubes. PBMCs were purified using a Ficoll-Histopaque (Fisher Scientific, NH) gradient. Cells were preserved in 90% fetal bovine serum (Sigma-Aldrich, MO) and 10% dimethyl sulfoxide (DMSO, Sigma-Aldrich, MO) and stored in liquid nitrogen.

### 4.3 Genomic DNA extraction and immunosequencing of TCR repertoires

Cryopreserved PBMC samples were thawed as described previously (8). Genomic DNA was isolated from 3 – 5 million PBMCs per participant for Immunosequencing using the QIAamp DNA Mini Kit (Qiagen). An average of 24.4±11.2 μg total DNA was extracted from 3 – 5 million PBMCs per participant. The isolated and purified genomic DNA was then used for deep immunosequencing of the CDR3 TCRB performed by Adaptive Biotechnologies using the ImmunoSeq Assay (38, 39).

### 4.4 HLA and KIR haplotyping from next-generation total RNA sequencing data

Total RNA was isolated from whole blood stabilized in PAXGene blood RNA tubes. Total RNA was used as input for total RNA-seq libraries using the Illumina Stranded Total RNA Prep with Ribo-Zero Plus and IDT^®^ for Illumina^®^ RNA UD Indexes Set A, Ligation (96 Indexes, 96 Samples). Sequencing libraries were assessed for size distribution and absence of adapter dimers using the Fragment Analyzer 5300 and quantified by qPCR on the Roche Light Cycler 480 Instrument II. Sequencing libraries were pooled and sequenced on an Illumina NovaSeq 6000 System using a S4 flowcell with a 200-cycle SBS kit. Paired-end FASTQ files, generated from total RNA sequencing, were used as the input for the T1K computational method (104). We utilized a default alignment similarity threshold of 97% to avoid low-quality reads and false-positive read assignments. T1K selected the allele with the highest abundance and filtered other alleles with abundances less than 15% of the dominant allele.

### 4.5 Analysis of TCRB sequences for SARS-CoV-2 specificity

The VDJdb with sequence inputs up to 6/21/2024 were included in analysis. The ImmuneCODE database contains a large-scale of TCRB sequences and matching SARS-CoV-2 epitopes based on TCR specificity measured using Multiplexed assay for Identification of T cell Receptor Antigen Specificity (MIRA) and ImmunoSEQ (Adaptive Biotechnologies) (38–40). The ImmuneCODE database (Release002, Adaptive Biotechnologies) was divided into MHC-I and MHC-II, in which the CD8 TCR sequences were dominant, and used for the focus of analysis in this study. In order to improve the comprehensiveness and precision of the database, human CD8 TCRB sequences from other resources were also included (**Table 2**). CD8 TCRB sequences in the ImmuneCODE database that were also found from pre-pandemic data were not included for SARS-CoV-2 specificity analysis. R package LymphoSeq (version 1.14, https://github.com/davidcoffey/LymphoSeq) was mainly used for the analysis (46). Specifically, productive sequences were identified using function “productiveSeq”. Clonality was calculated with the function “clonality”, as the inverted normalized Shannon entropy, which is the ratio between the frequencies of all productive sequences and the logarithm of the total number of unique clonotypes. CD8 TCRB specific for an antigen were identified with the criteria that the V and J genes, and CDR3β amino acid sequences all match a recorded CD8 sequence from the databases. The frequency of SARS-CoV-2-specific CD8 TCRB is considered as the counts of SARS-CoV-2-specific CD8 TCRB out of the total counts of all productive sequences within each participant.

In addition, R package VennDiagram (version 1.7.3, https://cran.r-project.org/web/packages/VennDiagram/index.html) was used to analyze and plot the overlapped TCRB sequences or epitopes (105). R package circlize (version 0.4.16, https://github.com/jokergoo/circlize/) was used to visualize the V and J association among TCRB sequences (106). R package ggseqlogo (Version 0.2, https://github.com/omarwagih/ggseqlogo) was used to plot amino acids in the CDR3β region (107).

### 4.6 Analysis of TCRB clusters

The TCRB sequences recognizing the same peptide were grouped. The CDR3β amino acid sequence, V and J genes, and counts were extracted and formatted as required and uploaded to the GLIPH2 server. GLIPH2 version 2.0 and CD8 on the server were selected, and CDR3α was not used for the prediction.

### 4.7 Analysis of CD4 TCRB sequences for SARS-CoV-2 specificity

The analysis of CD4 TCRB sequences utilized the same database resources as the CD8 analysis, albeit using the MHC-II portion of the aforementioned ImmuneCODE database. Database and participants files were loaded onto separate folders and placed within an umbrella folder where a Python script was written for the CD4 analysis. The Python packages named pandas (version 2.2, https://github.com/pandas-dev/pandas) and tcrdist3 (version 0.2.2, https://github.com/kmayerb/tcrdist3) were primarily used to perform the data transformations necessary for this analysis. Clonality was calculated with a locally defined function and defined as the inverted normalized Shannon entropy, which is the ratio between the frequencies of all productive sequences and the logarithm of the total number of unique clonotypes. CD4 TCRB specific for an antigen were identified with the criteria that the V and J genes, and CDR3β amino acid sequences all match a recorded CD4 sequence from the databases. The frequency of SARS-CoV-2-specific CD4 TCRB is considered as the counts of SARS-CoV-2-specific CD4 TCRB out of the total counts of all productive sequences within each participant.

### 4.8 Statistical analysis

In addition to the analyses mentioned above, if not specified, data were exported from R and then plotted using GraphPad Prism 10 (GraphPad Software). Two-group test significance levels were calculated using Mann-Whitney analysis. Multi-group test significance levels were calculated using Kruskal-Wallis rank sum test. Correlation coefficients and significance levels were calculated using Spearman rank correlation. A P value < 0.05 was considered statistically significant.

## Data Availability

The datasets presented in this article are not readily available because they include sequencing data from active duty military members that is restricted through the Infectious Diseases Clinical Research Program. Requests to access the datasets should be directed to Dr. Allison Malloy (allison.malloy@usuhs.edu).

## Group member of EPICC COVID - 19 Cohort Study Group

The following members for the EPICC COVID - 19 Cohort Study Group have met criteria for group authorship:

Naval Medical Center San Diego, San Diego, CA: D. Larson

United States Air Force School of Aerospace Medicine, Dayton: A. Fries

Walter Reed National Military Medical Center, Bethesda, MD: A. Ganesan

William Beaumont Army Medical Center, El Paso, TX: R. Mody

Brooke Army Medical Center, Fort Sam Houston, TX: J. Cowden; M. Darling; S. DeLeon; Maj D. Lindholm; A. Markelz; K. Mende; S. Merritt; T. Merritt; N. Turner; T. Wellington

Carl R. Darnall Army Medical Center, Fort Hood, TX: S. Bazan; P.K Love

Fort Belvoir Community Hospital, Fort Belvoir, VA: N. Dimascio-Johnson; N. Elnahas; E. Ewers; K. Gallagher; C. Glinn; U. Jarral; D. Jennings; D. Larson; K. Reterstoff; A. Rutt; A. Silva; C. West

Henry M. Jackson Foundation, Inc., Bethesda, MD: P. Blair; J. Chenoweth; D. Clark

Madigan Army Medical Center, Joint Base Lewis McChord, WA: J. Bowman; S. Chambers; C. Colombo; R. Colombo; C. Conlon; K. Everson; P. Faestel; T. Ferguson; L. Gordon; S. Grogan; S. Lis; M. Martin; C. Mount; D. Musfeldt; D. Odineal; M. Perreault; W. Robb-McGrath; R. Sainato; C. Schofield; C. Skinner; M. Stein; M. Switzer; M. Timlin; S. Wood

Naval Medical Center Portsmouth, Portsmouth, VA: S. Banks; R. Carpenter; L. Kim; K. Kronmann; T. Lalani; T. Lee; A. Smith; R. Smith; R. Tant; T. Warkentien

Naval Medical Center San Diego, San Diego, CA: C. Berjohn; S. Cammarata; N. Kirkland; D. Libraty; R. Maves; G. Utz

Tripler Army Medical Center, Honolulu, HI: C. Bradley; S. Chi; R. Flanagan; A. Fuentes; M. Jones; N. Leslie; C. Lucas; C. Madar; K. Miyasato; C. Uyehara

Uniformed Services University of the Health Sciences, Bethesda, MD: H. Adams; B. Agan; L. Andronescu; A. Austin; B. Barton, D. Becher, C. Broder; T. Burgess; C. Byrne; K Chung; J. Davies; C. English; N. Epsi; C. Fox; M. Fritschlanski; A. Hadley; P. Hickey; E. Laing; C. Lanteri; LTC J. Livezey; A. Malloy; A. Michel, R. Mohammed; C. Morales; P. Nwachukwu; C. Olsen; E. Parmelee; S. Pollett; S. Richard; J. Rothenberg, J. Rozman; J. Rusiecki; D. Saunders; E. Darcy; M. Sanchez; A. Scher; M. Simons; A. Snow; K. Telu; D. Tribble; M. Tso; L. Ulomi; M. Wayman, N. Hockenbury

United States Air Force School of Aerospace Medicine, Dayton, OH: T. Chao; R. Chapleau; M. Christian; A. Fries; C. Harrington; V. Hogan; S. Huntsberger; K. Lanter; E. Macias; J. Meyer; S. Purves; K. Reynolds; J. Rodriguez; C. Starr

United States Coast Guard, Washington, DC: J. Iskander; I. Kamara

Womack Army Medical Center, Fort Bragg, NC: B. Barton; D. Hostler; J. Hostler; K. Lago; C. Maldonado; J. Mehrer

William Beaumont Army Medical Center, El Paso, TX: T. Hunter; J. Mejia; R. Mody; J. Montes; R. Resendez; P. Sandoval

Walter Reed National Military Medical Center, Bethesda, MD: I. Barahona; A. Baya; A. Ganesan; N. Huprikar; B. Johnson

Walter Reed Army Institute of Research, Silver Spring, MD: S. Peel

## Author disclaimer

Several of the authors are active-duty service members or employees of the U.S. Government. This work was prepared as part of their official duties. Title 17 U.S.C. §105 provides that ‘Copyright protection under this title is not available for any work of the United States Government.’ Title 17 U.S.C. §101 defines a U.S. Government work as a work prepared by a military service member or employee of the U.S. Government as part of that person’s official duties. The contents of this publication are the sole responsibility of the author(s) and do not necessarily reflect the views, opinions, or policies of Uniformed Services University of the Health Sciences (USU); National Institutes of Health or the Department of Health and Human Services; the Henry M. Jackson Foundation for the Advancement of Military Medicine, Inc.; the Department of Defense (DoD); the U.S. Army Medical Department; the U.S. Army Office of the Surgeon General; the Departments of the Army, Navy, or Air Force; Brooke Army Medical Center; Walter Reed National Military Medical Center; Naval Medical Center San Diego; and Madigan Army Medical Center. Mention of trade names, commercial products, or organizations does not imply endorsement by the U.S. Government. The investigators have adhered to the polices for protection of human subjects as prescribed in 45 CFR 46.
