# Supplementary material for: Patterns of restricted TCR usage following SARS-CoV-2 vaccination and severe disease
Source: Front Immunol. 2025 Oct 2;16:1576903. doi: 10.3389/fimmu.2025.1576903 (PMC12528067; doi:10.3389/fimmu.2025.1576903)
Supplement: Supplementary file 1 [file Presentation1.pptx]

## Slide 1
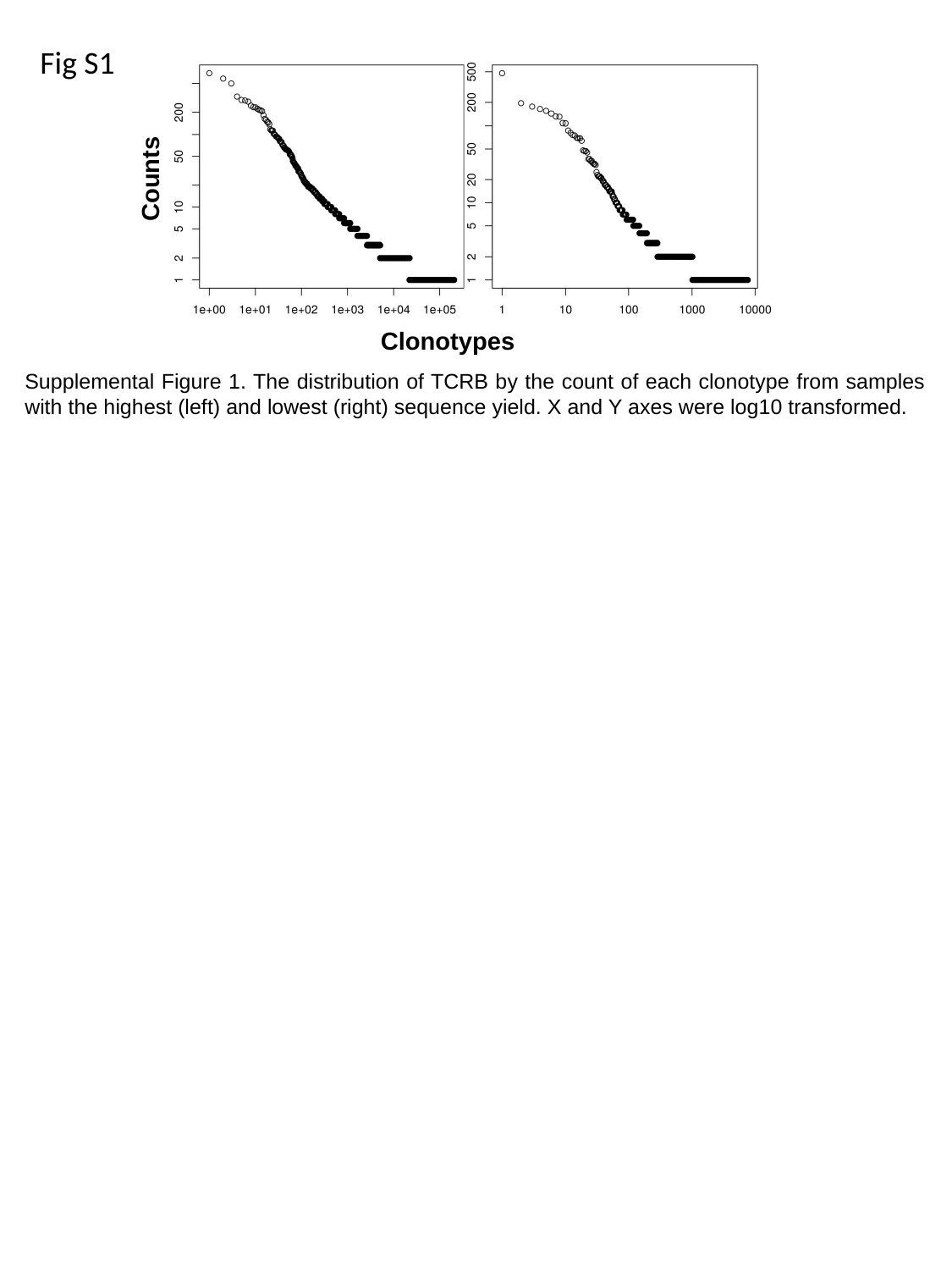

Fig S1
Counts
Clonotypes
Supplemental Figure 1. The distribution of TCRB by the count of each clonotype from samples with the highest (left) and lowest (right) sequence yield. X and Y axes were log10 transformed.

## Slide 2
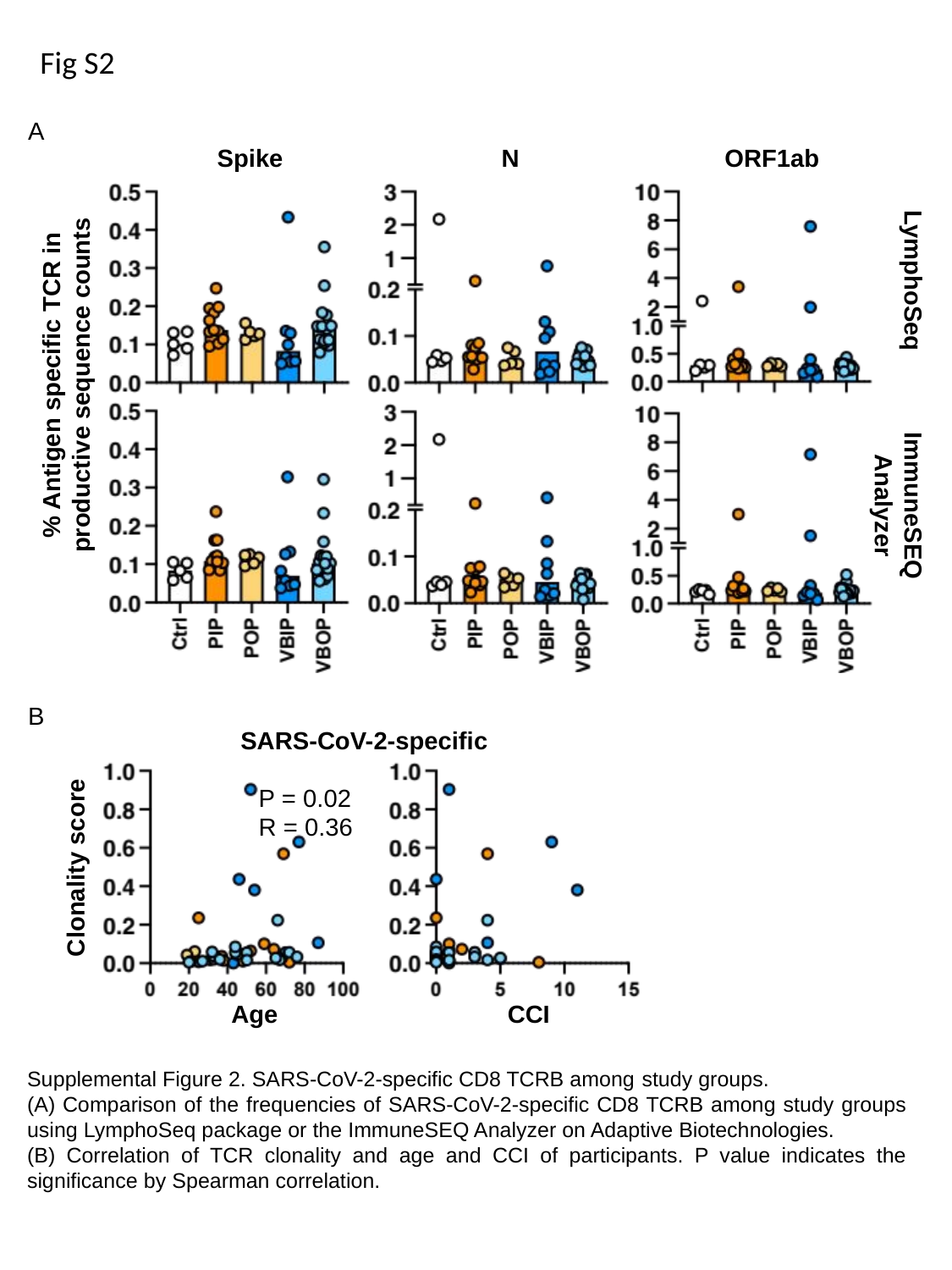

Fig S2
A
Spike
N
ORF1ab
LymphoSeq
% Antigen specific TCR in productive sequence counts
ImmuneSEQ Analyzer
B
SARS-CoV-2-specific
P = 0.02
R = 0.36
Clonality score
Age
CCI
Supplemental Figure 2. SARS-CoV-2-specific CD8 TCRB among study groups.
(A) Comparison of the frequencies of SARS-CoV-2-specific CD8 TCRB among study groups using LymphoSeq package or the ImmuneSEQ Analyzer on Adaptive Biotechnologies.
(B) Correlation of TCR clonality and age and CCI of participants. P value indicates the significance by Spearman correlation.

## Slide 3
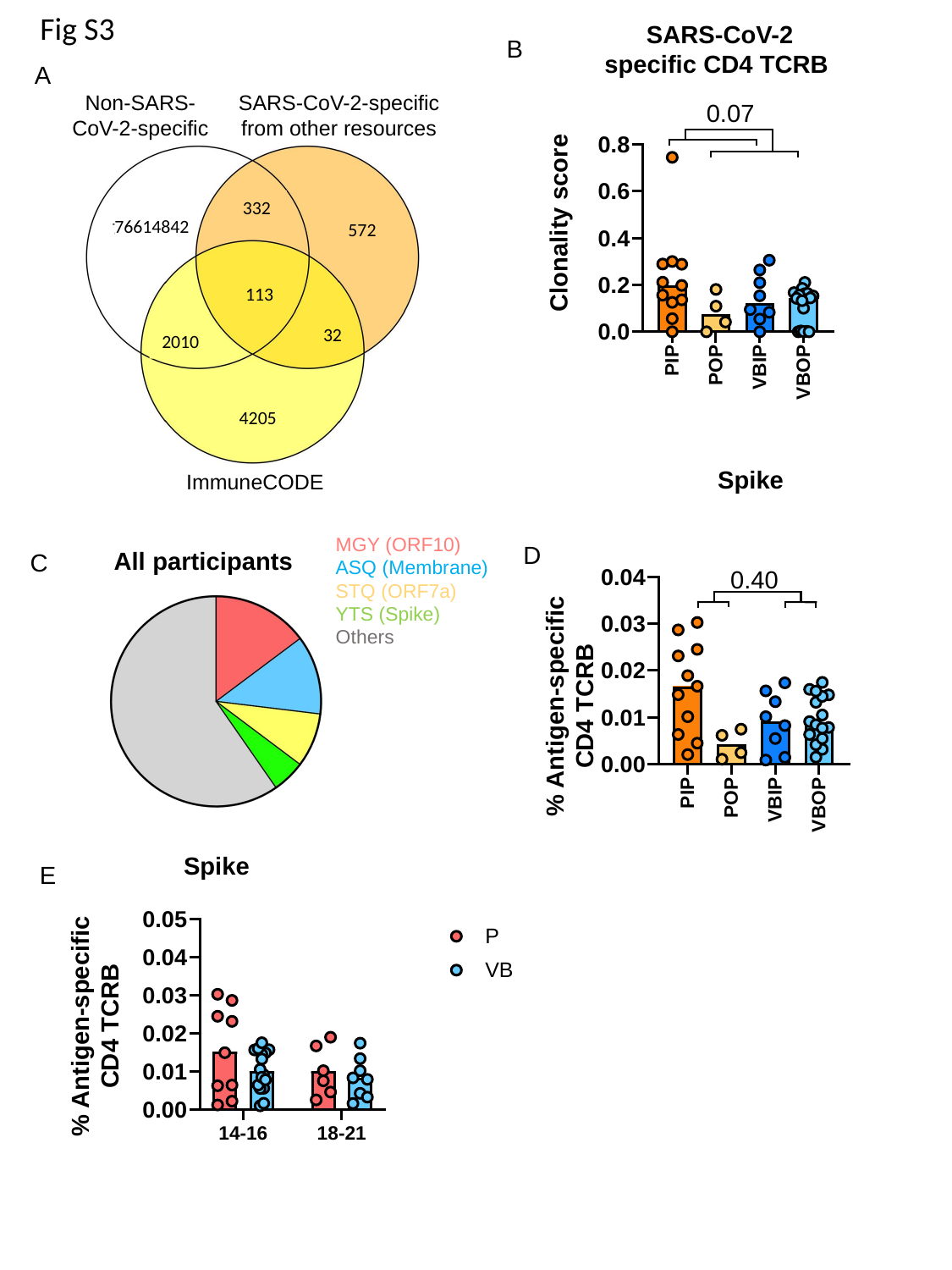

Fig S3
SARS-CoV-2 specific CD4 TCRB
B
Clonality score
A
Non-SARS-CoV-2-specific
SARS-CoV-2-specific from other resources
332
76614842
572
113
32
2010
4205
ImmuneCODE
0.07
Spike
D
0.40
% Antigen-specific CD4 TCRB
MGY (ORF10)
ASQ (Membrane)
STQ (ORF7a)
YTS (Spike)
Others
All participants
C
Spike
% Antigen-specific CD4 TCRB
E

## Slide 4
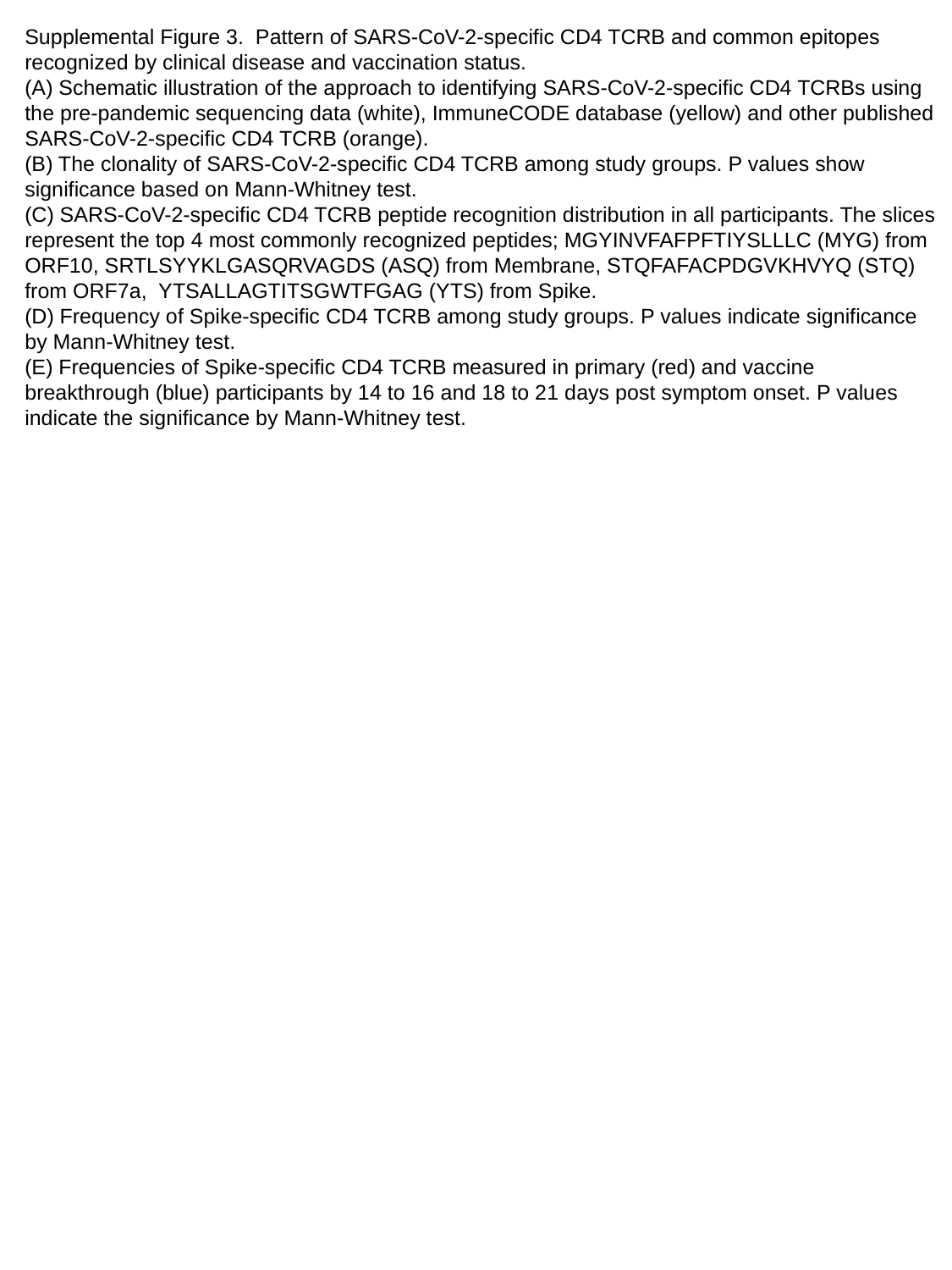

Supplemental Figure 3.  Pattern of SARS-CoV-2-specific CD4 TCRB and common epitopes recognized by clinical disease and vaccination status.
(A) Schematic illustration of the approach to identifying SARS-CoV-2-specific CD4 TCRBs using the pre-pandemic sequencing data (white), ImmuneCODE database (yellow) and other published SARS-CoV-2-specific CD4 TCRB (orange).
(B) The clonality of SARS-CoV-2-specific CD4 TCRB among study groups. P values show significance based on Mann-Whitney test.
(C) SARS-CoV-2-specific CD4 TCRB peptide recognition distribution in all participants. The slices represent the top 4 most commonly recognized peptides; MGYINVFAFPFTIYSLLLC (MYG) from ORF10, SRTLSYYKLGASQRVAGDS (ASQ) from Membrane, STQFAFACPDGVKHVYQ (STQ) from ORF7a, YTSALLAGTITSGWTFGAG (YTS) from Spike.
(D) Frequency of Spike-specific CD4 TCRB among study groups. P values indicate significance by Mann-Whitney test.
(E) Frequencies of Spike-specific CD4 TCRB measured in primary (red) and vaccine breakthrough (blue) participants by 14 to 16 and 18 to 21 days post symptom onset. P values indicate the significance by Mann-Whitney test.

## Slide 5
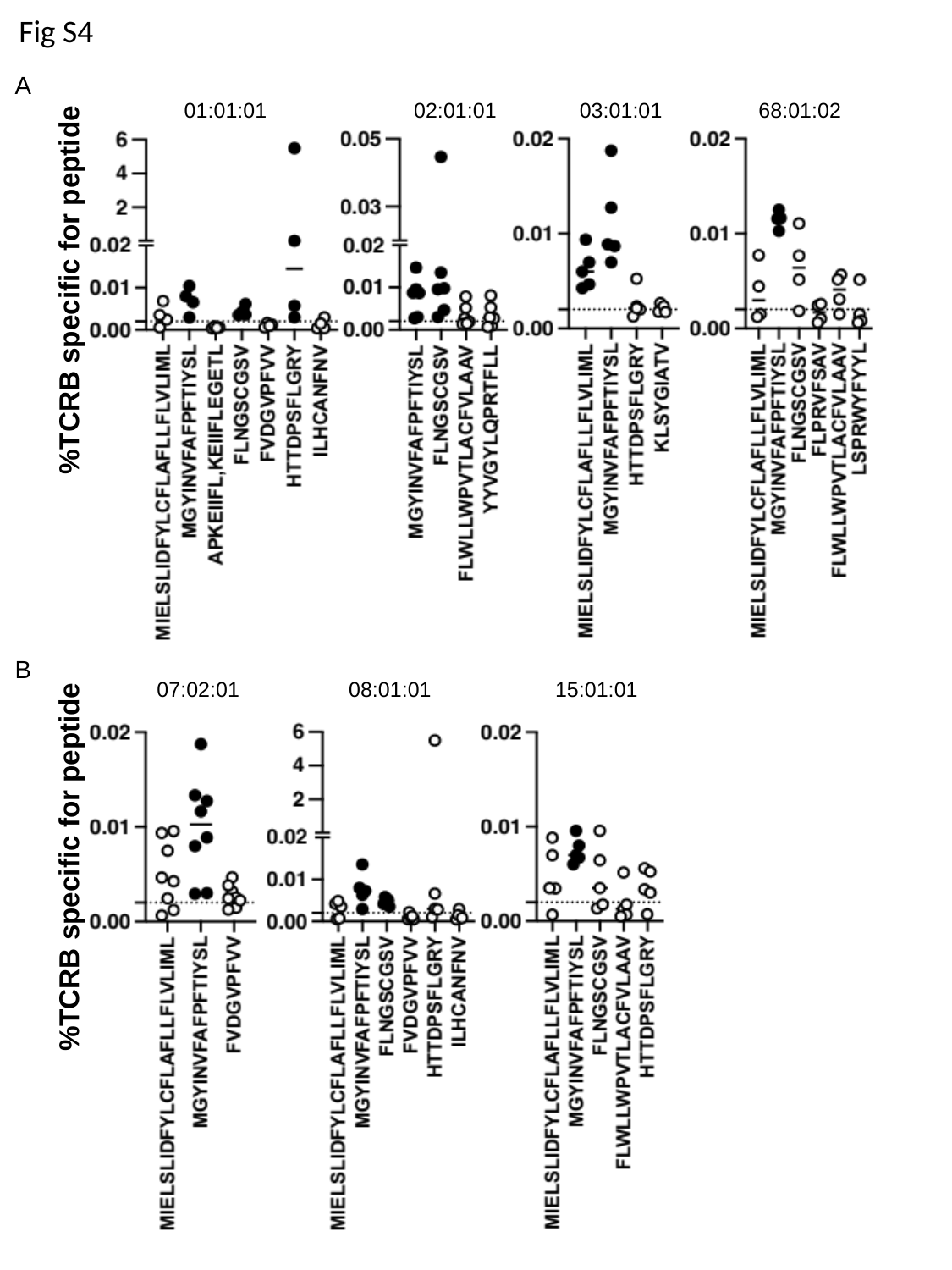

Fig S4
A
01:01:01
02:01:01
03:01:01
68:01:02
%TCRB specific for peptide
B
08:01:01
15:01:01
07:02:01
%TCRB specific for peptide

## Slide 6
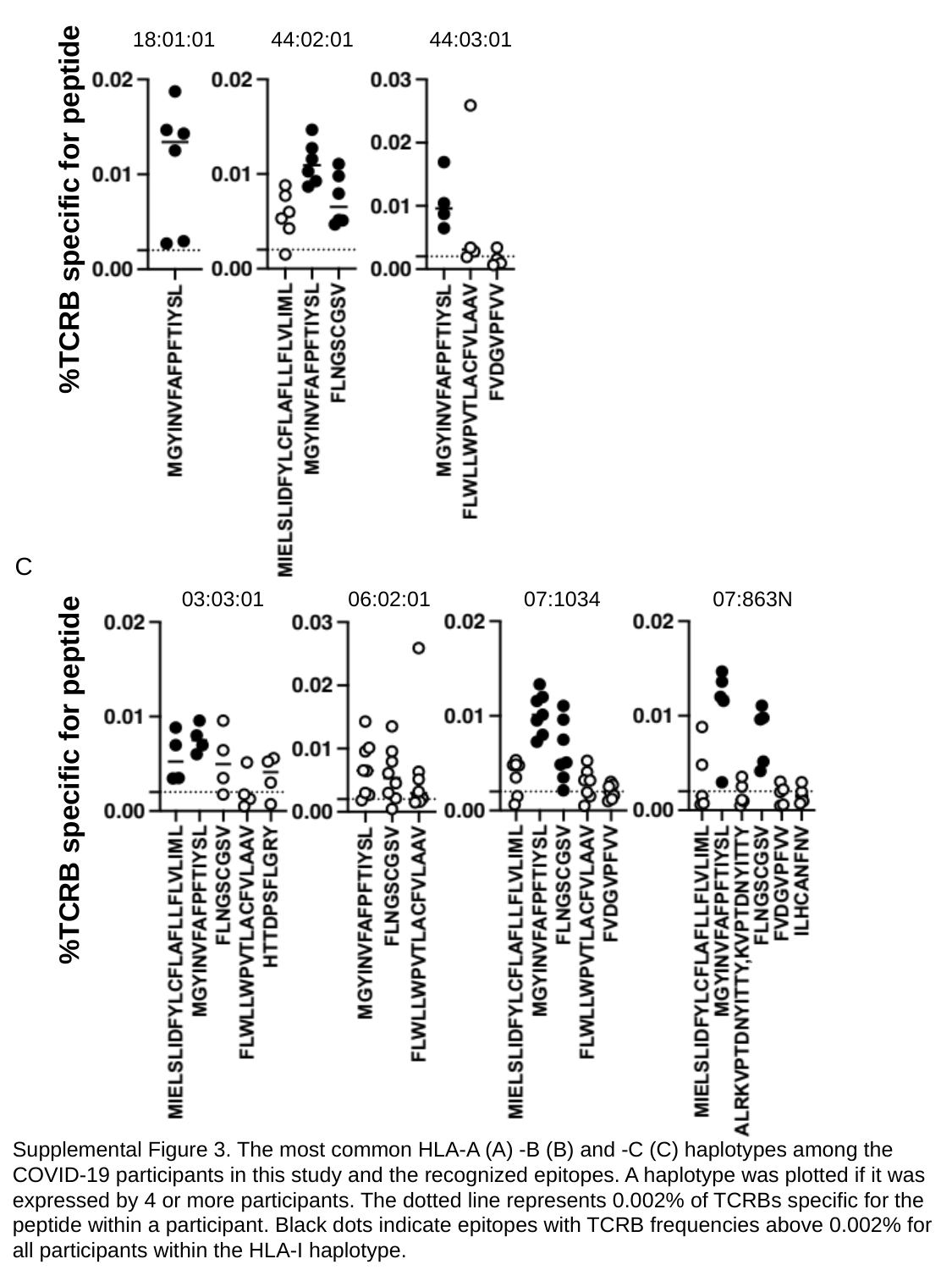

18:01:01
44:02:01
44:03:01
%TCRB specific for peptide
C
07:863N
03:03:01
06:02:01
07:1034
%TCRB specific for peptide
Supplemental Figure 3. The most common HLA-A (A) -B (B) and -C (C) haplotypes among the COVID-19 participants in this study and the recognized epitopes. A haplotype was plotted if it was expressed by 4 or more participants. The dotted line represents 0.002% of TCRBs specific for the peptide within a participant. Black dots indicate epitopes with TCRB frequencies above 0.002% for all participants within the HLA-I haplotype.
